# Controllable hybrid plasmonic integrated circuit

**DOI:** 10.1038/s41598-023-37228-4

**Published:** 2023-06-20

**Authors:** Maryam Khodadadi, Seyyed Mohammad Mehdi Moshiri, Najmeh Nozhat, Mohsen Khalily

**Affiliations:** 1 Department of Electrical Engineering, Shiraz University of Technology, Shiraz, Iran; 2 5G & 6G Innovative Centers (5GIC & 6GIC), Institute for Communication Systems (ICS), University of Surrey, Guildford, UK

**Keywords:** Nanophotonics and plasmonics, Optical properties and devices

## Abstract

In this paper, a controllable hybrid plasmonic integrated circuit (CHPIC) composed of hybrid plasmonic waveguide (HPW)-based rhombic nano-antenna, polarization beam splitter, coupler, filter, and sensor has been designed and investigated for the first time. In order to control the power into a corresponding input port, a graphene-based 1 × 3 power splitter with switchable output has been exploited. The functionality of each device has been studied comprehensively based on the finite element method and the advantages over state-of-the-art have been compared. Moreover, the effect of connection of CHPIC to the photonic and plasmonic waveguides has been studied to exhibit the capability of variety excitation methods of the CHPIC. Furthermore, the performance of the proposed CHPIC connected to inter/intra wireless transmission links has been investigated. The wireless transmission link consists of two HPW-based nano-antennas as transmitter and receiver with the maximum gain and directivity of 10 dB and 10.2 dBi, respectively, at 193.5 THz. The suggested CHPIC can be used for applications such as optical wireless communication and inter/intra-chip optical interconnects.

## Introduction

Photonic integrated circuits (PICs) bring unprecedented and significant promise for realizing prominent identifiable features such as low-cost and scaled optical solutions in wireless optical communication, sensing, computation, filtering, spectroscopy, beam steering, etc.^1^. The downscaling and integration of discrete components such as bends, ring resonators, splitters, couplers, sensors, and antennas hold new concept knobs and functionalities that are inaccessible in conventionally reported photonic devices^2^. Moreover, typical discrete plasmonic and photonic waveguide components cannot concurrently support ultra-low propagation loss, fast and efficient all-optical functionalities, and overcome their bottlenecks such as diffraction limit requirement, respectively, which result in the necessity of finding feasible solutions to eliminate these issues^3^.

The idea of utilizing HPW components is one of the most disruptive technology enablers to address most current limitations such as suffering from excessive ohmic losses and facilitate the functional standards of PICs to offer proposing several discrete components, which is suitable for the miniaturization of integrated circuit design. Many material systems and different configurations have been investigated and adopted for hybrid plasmonic components of integrated circuits^4^. Good material examples to design ultra-compact, broadband, and ultra-low losses hybrid plasmonic integrated circuits (HPICs) include silicon (Si)^5^, indium phosphide (InP)^6^, silicon nitride (SiN_x_)^7^, gallium arsenide (GaAs)^8^, aluminum nitride (AlN)^9^, silicon carbide (SiC)^10^, and hydrogen silsesquioxane (HSQ) that are highly attractive for a variety of integrated optical applications^11^. HSQ is heavily utilized for high-resolution electron-beam lithography (EBL)^12^ of photonic devices and is fully compatible with most materials and fabrication processes. In principle, it brings two major advantages of high integration density and compatibility to complementary metal-oxide semiconductor (CMOS) technology. In addition, its almost identical refractive index facilitates the adaptation of existing silicon-on-insulator (SOI) design and manufacturing process of HPICs.

Also, HPWs are a merger of plasmonic and photonic counterparts, which provide ultra-tight confinement, long range propagation length, and support hybrid plasmonic modes. Therefore, they are rapidly revolutionizing a broad range of applications from its traditional uses in guiding transverse magnetic (TM) mode to emerging fields such as optical beam steering, wireless optical communication, sensing, computation, filtering, bending, splitting, and radiation of optical signals for their basic functions as an indispensable components in optical communication systems^2,13,14^.

The future of integrated circuits will require wireless chip-to-chip communication to overcome the bottlenecks caused by wired connections. The dispensable part of each wireless communication system is wideband antennas with compact size, ultra-high gain and efficiency, which allow high wireless data capacity of several tens of terabits per second^15^. Therefore, future of advanced chip-to-chip communication is more related to optimize wireless transmission link, which shows the importance of proposing high performance nano-antennas.

In this paper, the idea of long-range CHPIC has been realized, which includes indispensable optical HPW-based components such as nano-antenna, polarization beam splitter, coupler, sensor, and filter. In addition, an integrated plasmonic graphene-based 1 × 3 power splitter with switchable output has been designed. Furthermore, the performance of two CHPIC configurations designed by interconnection of these discrete components on the same and different chips has been proposed and evaluated. In order to verify the performance of the single mode desired HPW, its propagation features have been studied numerically and compared with the theoretical method of transfer matrix theory. Moreover, the functionality of each component has been investigated discretely and its performance has been optimized when it is embedded in the proposed CHPIC. To show the superiorities of all designed components, they have been compared with other published works.

The hybrid plasmonic rhombic nano-antenna (HPRNA) with utilizing the novel idea of dielectric director has been proposed to design an inter/intra wireless transmission link to degrade the sophisticated communication of different layers of a chip. Besides, the performance of the wireless link has been surveyed based on the finite element method (FEM) and Friis equation to confirm its appropriate functionality. Since the proposed CHPIC excitation may not be accomplished directly by the laser and the information is transmitted to the CHPIC input by means of plasmonic or photonic waveguide, the impact of coupling between plasmonic or dielectric waveguide and CHPIC has been studied. The obtained results reveal that the connection between CHPIC and photonic waveguide is more efficient than plasmonic one. The proposed CHPIC can be fabricated utilizing standard CMOS technology and EBL technique, and therefore can be integrated with other elements in an opto-electronic circuit.

## Hybrid plasmonic waveguide

**Figure 1:**
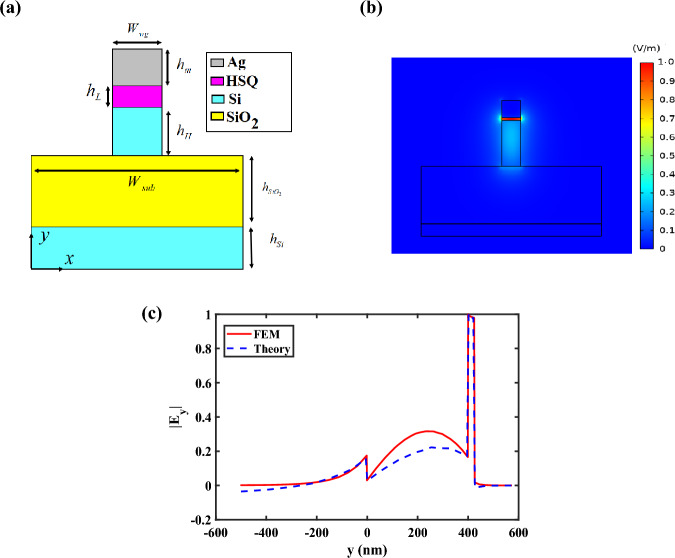
(**a**) Schematic cross-sectional view of the single mode HPW. (**b**) Distribution of TM mode profile and (**c**) electric field amplitude along y direction when x = 0 at 1550 nm (193.5 THz).

The first step to design a CHPIC is selecting a single mode HPW that its schematic cross-sectional view is shown in Fig. 1a. The proposed HPW consists of three layers of silver (Ag), HSQ, and silicon (Si) with the thicknesses of *h*_*m*_ = 150 nm, *h*_*L*_ = 25 nm and *h*_*H*_ = 400 nm, respectively. These layers are placed on a substrate consisting of two layers of silica (SiO_2_) and Si with the thicknesses of $h_{{{\text{SiO}}_{2} }} = 500\;{\text{nm}}$ and $h_{{{\text{Si}}}} = 800\;{\text{nm}}$, respectively. These thicknesses are selected based on the technological challenges and limits to be compatible with the SOI technology in order to avoid complexity of fabrication process. Moreover, the Si substrate is a bulk material with the thickness on the order of hundreds of micrometers in PICs, which can be considered as an infinite layer in comparison to the desired wavelength of 1550 nm (193.5 THz)^16^. Consequently, the thickness of 800 nm is considered for the Si layer to reduce the computation time and memory requirements. Moreover, SiO_2_ layer is thick enough to prevent damage to the silicon wafer, which is one of the most common substances used to develop PICs. Also, the widths of HPW and substrate are *W*_*wg*_ = 170 nm and *W*_*sub*_ = 1600 nm, respectively. The relative permittivities of HSQ and SiO_2_ layers are $\varepsilon_{HSQ} = 1.96$ and $\varepsilon_{{{\text{SiO}}_{2} }} = 1.96$, respectively. Although frequency-dependent optical phenomena such as optical dispersion offer advantages in various optical devices like prisms, they are beyond the scope of our current investigation^17^. Hence, we have omitted the consideration of optical dispersion in our study due to its minimal impact on the results. The relative permittivities of Ag and Si layers are extracted from Johnson-Christy and Palik data^18,19^. It must be noted that silver is selected as a metal cap layer due to its smallest total damping rate (Γ = 0.02 eV), lowest loss, much better surface plasmon polariton (SPP) confinement at telecommunication wavelengths, highest SPP quality factor in visible and near-infrared regions, and low fabrication cost^20^. In addition, HSQ as an amorphous structure similar to silica with a good adhesion on silicon wafers, is a good candidate for the low-index layer of the proposed HPW due to some advantages including high uniformity, high etching resistance, high resolution, and minimum line edge roughness^21^.

Distribution of the electric field y-component (|E_y_|) of the fundamental quasi-TM mode at 1550 nm (193.5 THz) is depicted in Fig. 1b, which reveals that the excited TM mode can confine in the HSQ layer tightly. On top of that, as illustrated in Fig. 1c, the obtained |E_y_| from the analytic solution of transfer matrix theory^16^ is well-matched with the result based on FEM. The analytic approach to obtain the dispersion relation of the proposed HPW has been introduced completely in Section S1 in the Supplementary Information.

In the HPW, the wave propagation is supported by the interaction of both SPPs and dielectric waveguide modes. This waveguide consists of a metal layer sandwiched between two dielectric layers. When considering a high-power mode, we need to consider the behavior of the electromagnetic field within the waveguide. The mode confinement is primarily determined by the refractive indices of different layers. In the HPW, the lower refractive index layer typically corresponds to the dielectric layer adjacent to the metal layer, while the higher refractive index layer corresponds to the outer dielectric layer. The difference in refractive indices creates a refractive index contrast across the waveguide structure. Now, in the context of a high-power mode, it means that the intensity of the electromagnetic field is high within the waveguide. This high intensity is associated with a higher concentration of energy. The confinement of a mode depends on the refractive index contrast. In the case of HPW, the lower refractive index layer adjacent to the metal layer offers a higher refractive index contrast compared to the higher refractive index layer. This higher contrast leads to a stronger confinement of the mode within the lower refractive index layer. The confinement of mode within the lower refractive index layer is advantageous because the metal layer in the HPW offers a strong ability to confine and guide SPPs. By confining the high-power mode within the layer with the lower refractive index, we can take advantage of both the enhanced field confinement due to the SPPs and lower propagation loss associated with the dielectric layer^3,14^.

Extracting data from Fig. S1 in the Supplementary Information shows that the propagation length of the proposed HPW is 94.4 μm at 193.5 THz that indicates SPPs can propagate without sharp damping up to 94.4 μm. Therefore, final foot-print of the suggested CHPIC should not exceed 94 μm at 193.5 THz. On the other hand, one of the limiting factors to design CHPIC is related to the frequency spectrum of devices such as nano-antenna. As a result, in the desired frequency spectrum (180–220 THz), the minimum propagation length at the frequency of 220 THz should be considered as the maximum dimension of the designed CHPIC.

It is essential to mention that silver oxidation over time is a common issue as silver is prone to oxidation when exposed to air or other oxidizing agents. This oxidation can degrade the optical properties of the metal layer in the HPW, affecting its performance. To overcome this issue, several strategies can be employed^22^:

1. *Protective coating* Applying a protective coating on the silver layer can prevent direct exposure to the atmosphere, leading to reducing oxidation. Various thin films or coatings such as silicon nitride (Si_3_N_4_) or aluminum oxide (Al_2_O_3_) can serve as effective barriers against oxidation while maintaining the optical properties of the HPW.
2. *Encapsulation* Encapsulating the HPW structure within a protective environment such as a hermetically sealed package or a controlled atmosphere can limit the exposure of the silver layer to oxygen and moisture. This approach can significantly reduce oxidation and enhance the long-term stability of the HPW.
3. *Material selection* Considering alternative metals or alloys with higher resistance to oxidation such as gold or aluminium can be a viable option. These metals are less prone to oxidation and can provide improved long-term stability for the HPW.
4. *Cleanroom fabrication* Ensuring that the fabrication process of the HPW takes place in a controlled environment like a cleanroom can minimize the exposure of the silver layer to contaminants that may accelerate oxidation. Maintaining strict cleanliness standards during fabrication can help reduce the oxidation rate.

## Hybrid plasmonic rhombic nano-antenna with dielectric director

**Figure 2:**
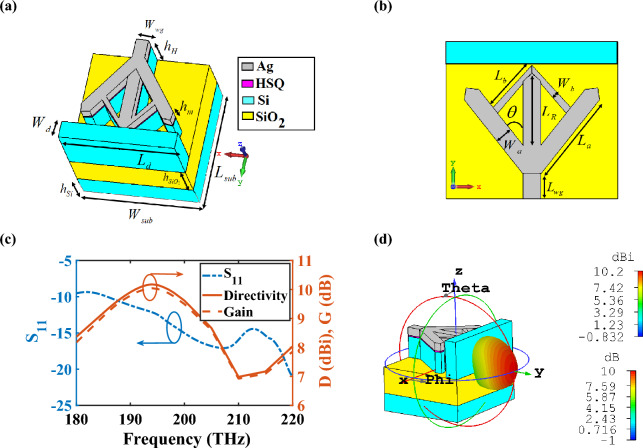
(**a**,**b**) The 3-dimensional (3D) scheme and top view of the HPRNA with dielectric director. (**c**) Reflection coefficient, gain and directivity spectra, and (**d**) 3D radiation directivity and gain pattern of HPRNA at 193.5 THz. The geometrical dimensions are *L*_*wg*_ = 250 nm, *L*_*a*_ = 915 nm, *L*_*b*_ = 554 nm, *L*_*R*_ = 829 nm, *W*_*a*_ = 170 nm, *W*_*b*_ = 70 nm, *W*_*wg*_ = 170 nm, *W*_*d*_ = 225 nm, *L*_*d*_ = 1600 nm, and $L_{sub} \times W_{sub} = 1560 \times 1600\;{\text{nm}}^{2}$. Other parameters are the same as Fig. 1a.

As it is mentioned in previous section, HPWs are capable to generate and propagate TM mode, where the field generated by the high-power mode is confined in the layer with the low refractive index, resulting in decreasing the propagation loss. As a result, utilizing HPW to design HPW-feed nano-antennas has attracted much attention to increase the gain and directivity of plasmonic nano-antennas and decrease the foot-print of dielectric antennas, which is suitable for optical wireless links at a chip-level scale. Therefore, in this section, the performance of a HPRNA with dielectric director is investigated, as demonstrated in Fig. 2a,b.

A single long HPW does not have high gain and directivity because of impedance mismatch between waveguide and free space, which leads to reflecting the propagated TM mode to the source. Up to now, to increase the impedance matching, the ideas of tapered and flared radiation parts are utilized. Hence, the concept of rhombic radiating part is used to compensate the low directivity and high side lobes and control the main beam angle of a single long HPW^23^. In such these nano-antennas, the aperture angle of radiation part plays an important role in controlling the main beam width, gain and direction of radiated pattern. If the flaring angle of the tapered opening is close to 90º, the mismatch between the flared part and free space increases, because it plays as an opening HPW, which results in decreasing the far-field performance of the proposed HPRNA drastically. Hence, the HPRNA dimensions should be optimized to obtain the desired directivity and gain for the suggested CHPIC. Based on the proposed formula in ref.^23^ for estimating the far-field performance of V-shaped HPW-feed nano-antenna versus the flared angle and using the optimization method and setting the goal of achieving the directivity of 10 dBi, the optimized angle of $\theta = 36^\circ$ is obtained. On the other hand, in order to optimize the thicknesses of layers, the intention of achieving a better compromise between the maximum propagation length and figure of merit (FOM) is employed. Consequently, to maintain this concept as the most critical feature of the suggested CHPIC, the thicknesses of layers have not been considered as one of the parameters to achieve the objective function in the optimization method. The length of the flared radiation part of the HPRNA is another effective parameter in controlling the gain and directivity of nano-antenna, which is chosen based on the idea in ref.^24^ and then the best length is obtained with the help of optimization method.

The CST Microwave Studio software is used to obtain the simulation results. The waveguide port is used to excite the proposed HPW. Also, to calculate the excited mode in the port, which should be perfectly matched with the waveguide modes inside the HPW to have a low reflection, the Eigenmode solver is utilized. In order to control the scattered light, it is essential to choose the open (add space) boundary condition. The HPW is meshed with 6,087,458 hexahedral mesh configurations based on setting the local mesh and activation of the adaptive hexahedral mesh refinement with convergence condition of 1% maximum energy variance between adjacent iterations to obtain the accurate computational results and ensure the validity of the used mesh. Mentioned conditions for meshing strategy is necessary because a very thin layer of graphene used in some devices, which will be discussed in the next sections, needs an appropriate mesh configuration in order to play its role.

In order to investigate the far-field characteristics of the proposed HPRNA with director, the return loss spectrum (S_11_) is shown in Fig. 2c. It can be seen that the impedance matching between the HPW and flared radiation part occurs in the frequency range of 185–220 THz. Therefore, it is possible to study the antenna performance in this interval because the gain and directivity of HPRNA are valid in this range. The 3D radiation pattern of HPRNA with Si director is plotted in Fig. 2d. It can be seen that a completely horizontal radiation pattern with 3 dB bandwidth of 43.5° and side lobe level of − 11.5 dB are attained, which is useful for point-to-point connections in wireless links and networks. In this regard, the far-field monitors are used in the desired frequency range to derive the far-field components of the HPW to attain gain, directivity, efficiency and radiation pattern from the calculated fields stored on the bounding box of the calculation domain, which is far away from the excitation source at the quarter of wavelength by performing the standard near-to-far field projections of the fields recorded for different frequencies. The resolution of 1° is chosen for the far-field radiation patterns. Utilizing the Si director improves the gain and bandwidth of the HPRNA. According to Fig. 2c, the bandwidth of the proposed HPRNA with dielectric director is 35 THz. Furthermore, the maximum gain and directivity are 10 dB and 10.2 dBi at 193.5 THz, respectively, with the efficiency of 95.5%.

There is an intuitive explanation for why the Si director can solve the tilted radiation pattern issue in the antenna design. When an electromagnetic wave is incident upon a dielectric material with a specific refractive index, the direction of the wave is bent or refracted at the interface between the two materials. This bending effect can be used to control the directionality of the radiation pattern emitted by the antenna. In the case of a tilted radiation pattern, the wavefronts of the electromagnetic radiation are not perpendicular to the surface of the antenna, leading to a non-uniform distribution of the radiated power in different directions. By introducing the Si director with a specific refractive index and shape, the wavefronts can be refracted to achieve a desired radiation pattern. In essence, the Si director acts as a lens for the electromagnetic radiation, focusing it in a particular direction and mitigating the tilted radiation pattern issue.

Since the proposed CHPIC excitation may not be done directly by the laser (input port) and the information is transmitted to the optical CHPIC input by means of plasmonic or photonic waveguides (dielectric waveguide), it is necessary to investigate the impact of coupling between plasmonic or dielectric waveguides and HPW on the far-field and impedance matching characteristics of the proposed HPRNA (See Section S2.1 in the Supplementary Information).

Finally, the performance of the suggested HPRNA with Si director in terms of radiation characteristics such as efficiency, gain, type of radiation pattern and fabrication possibility are discussed in Table S1 in the Supplementary Information.

## Wireless point-to-point inter link

**Figure 3:**
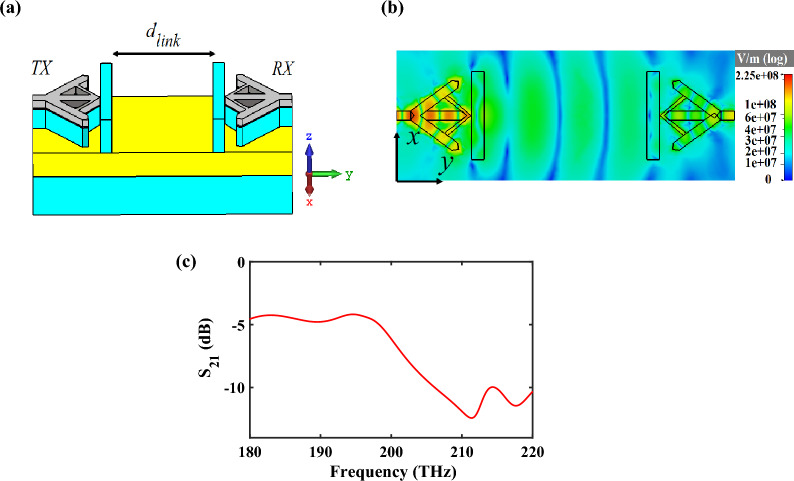
(**a**) 3D Schematic view of wireless link that consists of two HPRNAs as transmitter and receiver. (**b**) E-field distribution between two HPRNAs at 193.5 THz in the xy-plane and (**c**) the spectrum of the ratio of received power to transmitted power (S_21_) of the wireless link based on FEM simulation.

The considerable idea of proposing HPRNA is its ability to create an optical wireless link in order to transmit the electromagnetic power from transmitter antenna to receiver antenna because of its horizontal radiation pattern, high gain and efficiency. Consequently, the performance of the HPRNA is investigated as an on-chip wireless link, as plotted in Fig. 3a.

The ratio of the received power $\left( {P_{r} } \right)$ to the transmitted power $\left( {P_{t} } \right)$ can be calculated from the Friis equation^16^:

1 $$P_{r} |_{dB} - \, P_{t} |_{dB} = G_{r} \left( {\varphi_{r} ,\theta_{r} } \right)|_{dB} + G_{t} \left( {\varphi_{t} ,\theta_{t} } \right)|_{dB} + \, 10\log_{10} \left( {1 - \left| {\Gamma_{t} } \right|^{2} } \right)\left( {1 - \left| {\Gamma_{r} } \right|^{2} } \right) + 20\log_{10} \frac{{\left| {\hat{e}^{i} \cdot \hat{\ell }_{eff} } \right|}}{{\left( {{{4\pi n_{eff} d_{link} } \mathord{\left/ {\vphantom {{4\pi n_{eff} d_{link} } {\lambda_{0} }}} \right. \kern-0pt} {\lambda_{0} }}} \right)}}$$

where $10\log_{10} \left( {1 - \left| {\Gamma_{i} } \right|^{2} } \right)\,\,,\,\,\,i = t,\,\,r$ is transmitter/receiver impedance mismatch, $20\log_{10} \left| {\hat{e}^{i} \cdot \hat{\ell }_{eff} } \right|$ is polarization mismatch, and *d*_*link*_ is the distance between the transmitter and receiver antennas. Also, $\hat{e}^{i}$ and $\hat{\ell }_{eff}$ are complex unit vectors describing the polarization of the incident wave and the direction of the vector effective length of receiving antenna, respectively^16^. Based on the reciprocity theory, the gains of transmitter and receiver antennas are considered equal $\left( {G_{t,r} \left( {\varphi_{t,r} ,\theta_{t,r} } \right)} \right)$.

The electric field distribution of wireless transmission link (Fig. 3b) shows that the amplitude of the radiation power from the transmitter HPRNA is gradually reduced due to the propagation loss. Also, the guided power is recollected by the receiver and it is concentrated and transferred in the layer with the low refractive index. The attained results of the ratio of $P_{r}$ to $P_{t}$ by the Friis equation (Eq. (1)) and FEM simulation (Fig. 3c) are − 4.5 dB an − 4.98 dB, respectively, which the results are in good agreement with each other at the frequency of 193.5 THz.

## HPW-based polarization beam splitter

**Figure 4:**
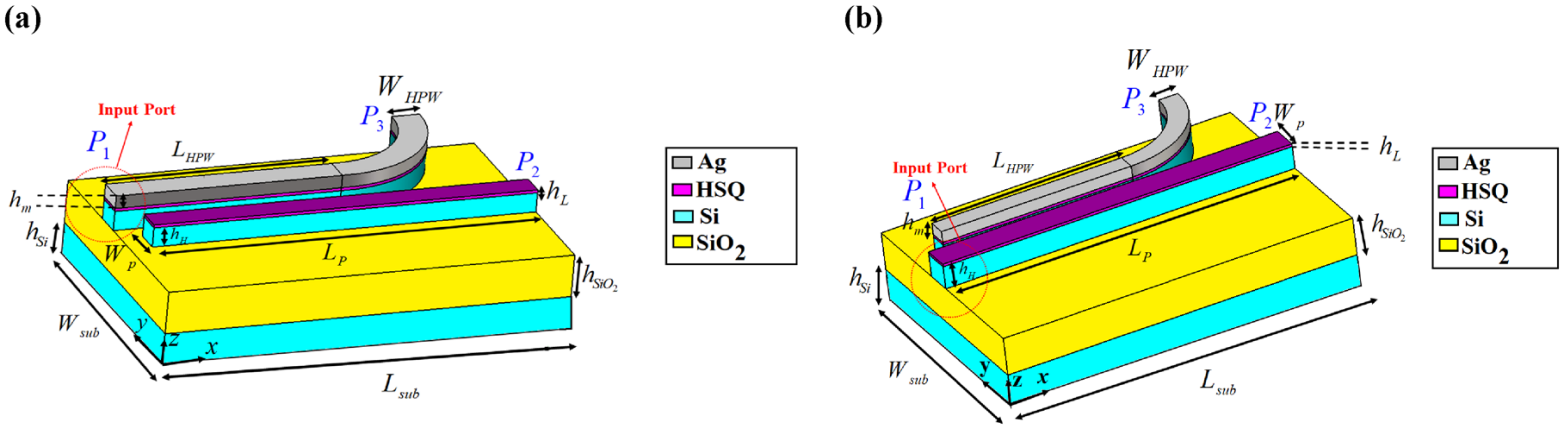
3D schematic views of the proposed PBS with (**a**) HPW and (**b**) dielectric waveguide input sections. The geometrical dimensions of PBS with HPW input section are *W*_*HPW*_ = 310 nm, *L*_*HPW*_ = 2535 nm, *W*_*sub*_ = 2900 nm, *L*_*sub*_ = 4550 nm, *L*_*p*_ = 4300 nm, *W*_*p*_ = 280 nm. The geometrical dimensions of PBS with dielectric waveguide input section are *W*_*HPW*_ = 270 nm, *L*_*HPW*_ = 4850 nm, *W*_*sub*_ = 3400 nm, *L*_*sub*_ = 4850 nm, *L*_*p*_ = 4850 nm, *W*_*p*_ = 400 nm.

One of the key components of optical CHPIC is polarization beam splitter (PBS), which separates transverse magnetic (TM) and transverse electric (TE) modes. Hence, the hybrid plasmonic mode of HPW is split into different ports and coupled to TE and TM modes to implement the polarization splitting function^25^. To date, many researches have been done to investigate different performances of PBSs based on various methods such as mode evolution and multimode interference^26^, or based on different structures such as Mach–Zehnder interferometer^27^, photonic crystal^28^, grating^29^, and directional coupler^30^. Studying the limitations and advantages of previous published works about PBSs reveals that directional coupling is the simplest and most flexible design. In this section, we will propose a compact HPW-based PBS that consists of a curved HPW and a bus dielectric waveguide. To design the PBS, two major points should be considered. First of all, as the HPRNA is excited by the HPW-feed line and the nano-antenna and PBS will be connected to each other in CHPIC, one of the PBS branches should be made by HPW. Secondly, the excitation of the proposed PBS can be done through the dielectric waveguide or HPW, which leads to the design of two different PBS structures. The 3D schematic views of two PBSs are shown in Fig. 4.

HPW and dielectric waveguide support both TE and TM modes, but in HPW, TM mode is concentrated in the low-index layer, whereas the conventional TE mode is concentrated in the high-index region. Supporting both TE and TM modes by HPW shows that this polarization diversity can be used to design TM- and TE-pass polarizer. Therefore, in the following the performance of PBS has been studied to set the specifications of switch mode (conversion of TE/TM to TM/TE modes).

**Figure 5:**
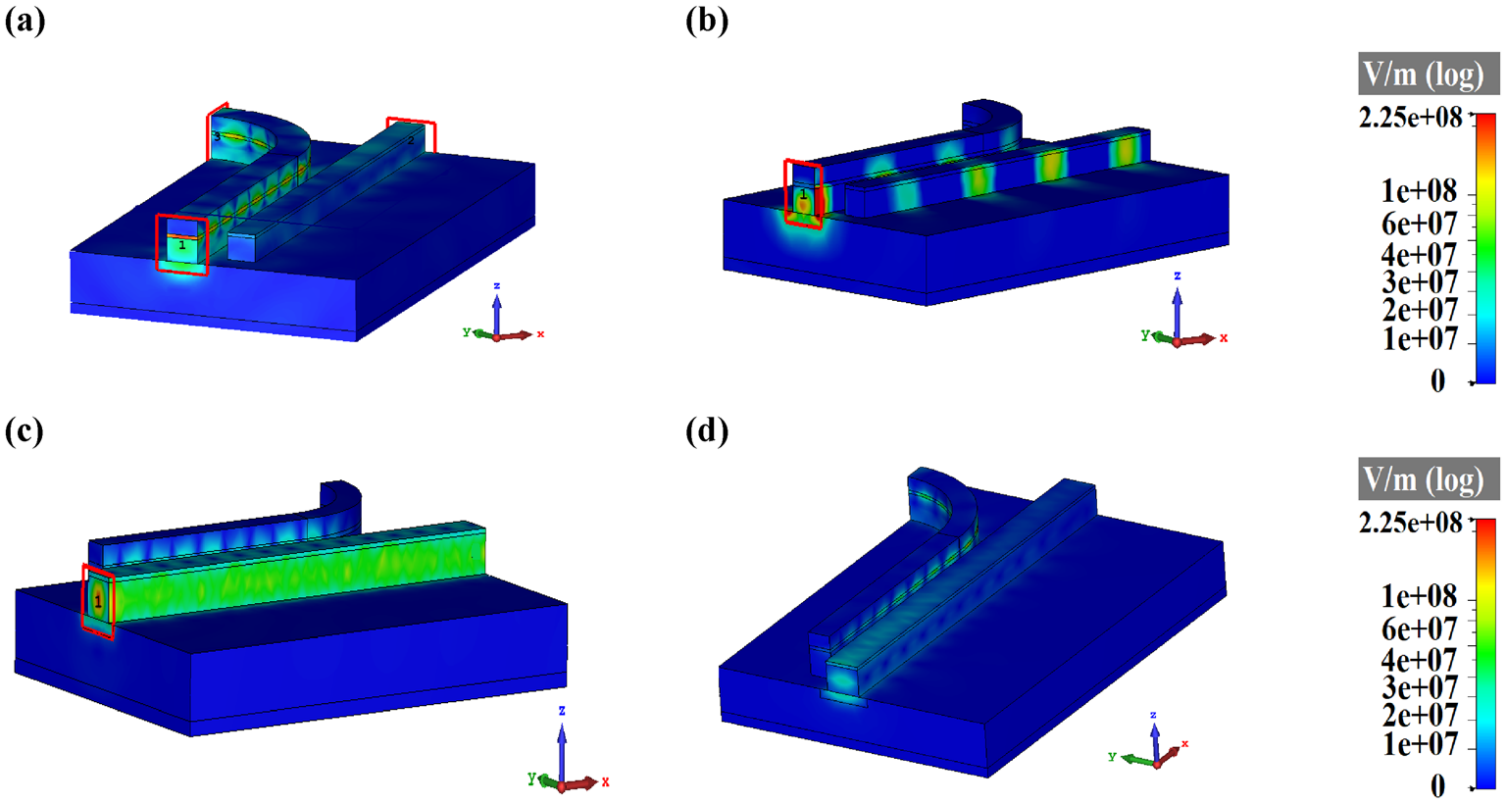
Electric field distributions of the PBS with HPW input section excited by (**a**) TM and (**b**) TE modes and PBS with dielectric waveguide input section excited by (**c**) TM and (**d**) TE modes.

As demonstrated in Fig. 5a, if the HPW is stimulated by the fundamental TM mode at 193.5 THz, the excited SPPs can pass through the HPW and a small amount of power is coupled to the dielectric waveguide. In return, the electric field distribution of Fig. 5b depicts that considerable power can be received at the dielectric waveguide output port by the excitation of HPW through the TE mode. In this way, the most guided TE mode is lost in the curved HPW and is coupled to dielectric waveguide, leading to the reduction of the reached power to the HPW output port. For further analysis, the transmission power spectra of the PBS with HPW input are illustrated in Fig. S6 in the Supplementary Information. It can be seen from Fig. S6a that for TM excitation, 74% of the received power is transmitted to the HPW output port and only 0.1% is transmitted to the dielectric waveguide output port. However, according to Fig. S6b, in the PBS with TE excitation mode only 36.15% of power is transmitted to the dielectric waveguide output port at the frequency of 193.5 THz. Therefore, the PBS with TM excitation has better performance.

Next, the PBS performance with the excitation through the dielectric waveguide is studied. The electric field distribution of Fig. 5c illustrates that by the excitation of PBS through the TM mode at the dielectric waveguide input port, most of the guided power is transmitted to the output through the dielectric waveguide and only low power is coupled to the low-index layer of the HPW. The transmission spectra of Fig. S7a in the Supplementary Information verify the predication and disclose that 95% of the input power is transmitted to the dielectric waveguide output port that compared to the TM-stimulated PBS through the HPW (Fig. 4a), it has better performance to pass TM optical power. Figure 5d demonstrates the electric field distribution of the PBS excited by TE mode at the dielectric waveguide input port. The TE excitation mode leads to the stimulation of fundamental TM mode of the proposed HPW. According to the transmission spectra of Fig. S7b in the Supplementary Information, the output transmission power of the HPW port is 76.85% at the frequency of 193.5 THz. Studies show that when the dielectric waveguide is excited by TE mode, the transmission power to the HPW output port is higher than the TE excitation of the HPW input port. Consequently, the proposed PBS of Fig. 4b with dietetic waveguide input section has better performance in comparison to the PBS with HPW one.

**Figure 6:**
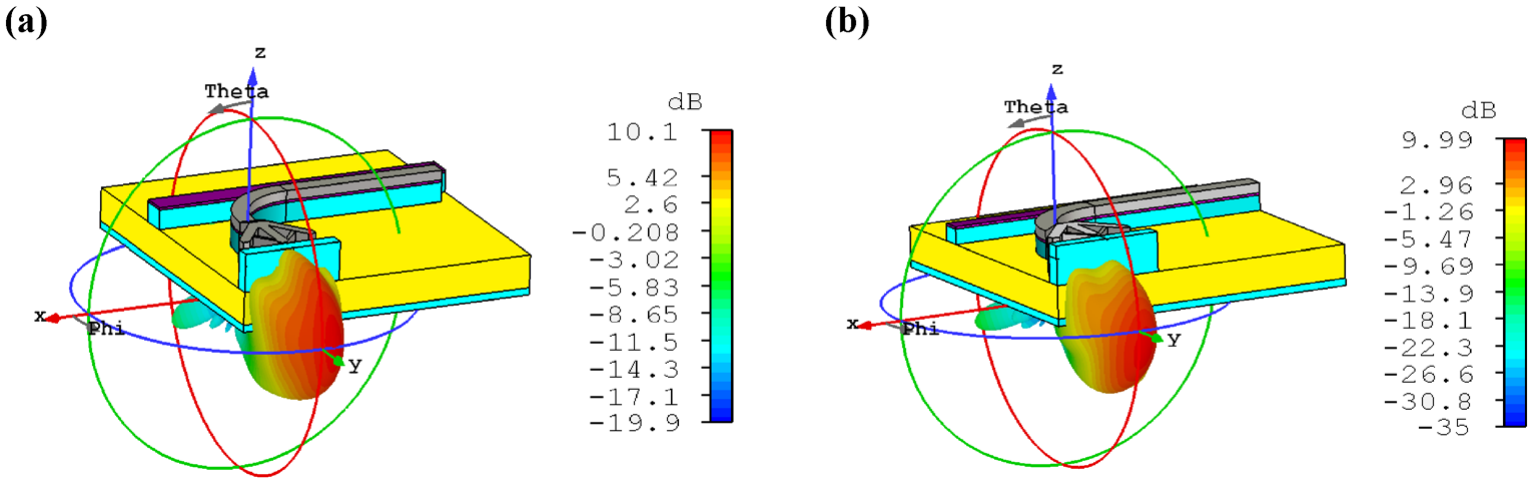
Radiation patterns of HPRNA and PBS connection with (**a**) dielectric waveguide input section and (**b**) HPW input section at 193.5 THz.

One of our concepts to design CHPIC is to investigate different ways to excite the HPRNA, which it can be stimulated by the guided TM mode through the HPW-based PBS. Therefore, it is necessary to study the far-field characteristics of the HPRNA connected to the PBS. According to the radiation patterns of Fig. 6, the excitation of HPRNA by the HPW-based PBS does not have a considerable impact on the antenna performance such as gain and direction of the main lobe.

Finally, to elucidate the device performance, the insertion loss (IL) and crosstalk (CT) of different modes for both proposed HPW-based PBS at the frequency of 193.5 THz have been calculated based on the following equations^25^ and they are listed in Table S2 in the Supplementary Information:

2 $${\text{IL}}_{TE}^{TE} = 10\log_{10} \left( {\frac{{P_{2}^{TE} }}{{P_{1}^{TE} }}} \right),\quad 10\log_{10} \left( {\frac{{P_{3}^{TE} }}{{P_{1}^{TE} }}} \right)$$

3 $${\text{IL}}_{TE}^{TM} = 10\log_{10} \left( {\frac{{P_{2}^{TM} }}{{P_{1}^{TE} }}} \right),\quad 10\log_{10} \left( {\frac{{P_{3}^{TM} }}{{P_{1}^{TE} }}} \right)$$

4 $${\text{IL}}_{TM}^{TM} = 10\log_{10} \left( {\frac{{P_{2}^{TM} }}{{P_{1}^{TM} }}} \right),\quad 10\log_{10} \left( {\frac{{P_{3}^{TM} }}{{P_{1}^{TM} }}} \right)$$

5 $${\text{IL}}_{TM}^{TE} = 10\log_{10} \left( {\frac{{P_{2}^{TE} }}{{P_{1}^{TM} }}} \right),\quad 10\log_{10} \left( {\frac{{P_{3}^{TE} }}{{P_{1}^{TM} }}} \right)$$

6 $${\text{CT}}_{TE}^{HPW} = 10\log_{10} \left( {\frac{{P_{3}^{TM} }}{{P_{3}^{TE} }}} \right),\quad {\text{CT}}_{TE}^{DW} = 10\log_{10} \left( {\frac{{P_{2}^{TM} }}{{P_{2}^{TE} }}} \right)$$

7 $${\text{CT}}_{TM}^{HPW} = 10\log_{10} \left( {\frac{{P_{2}^{TM} }}{{P_{2}^{TE} }}} \right),\quad {\text{CT}}_{TM}^{DW} = 10\log_{10} \left( {\frac{{P_{3}^{TE} }}{{P_{3}^{TM} }}} \right)$$

where $P_{{i\left( {1,2,3} \right)}}^{TE,TM}$ shows the power at port *i* with the excited TE or TM mode. For calculating ${\text{IL}}_{m(TE,TM)}^{n(TE,TM)}$, *m* and *n* represent the excited TM/TE mode at the input port and received TM/TE mode at the output port, respectively. Also, in order to obtain ${\text{CT}}_{m(TE,TM)}^{j(HPW,DW)}$, *j* depicts which waveguide (HPW/DW) is considered as the input port.

In order to determine the advantages of the proposed TE/TM polarizers, the structure specifications have been compared with other previous published works, as demonstrated in Table S3 in the Supplementary Information.

## Controllable HPW-based PBS

In order to design a dynamic and controllable HPW-based PBS, a multilayer graphene that consists of eight graphene sheets with the thickness of $\Delta _{g} = 0.5\;{\text{nm}}$ and a distance of 4 nm is placed inside the HSQ layer at the common boundary of HSQ and Si, as illustrated in Fig. S8a in the Supplementary Information. As the PBS with dielectric waveguide input port has better performance, only this design is investigated to bring forth of a controllable HPW-based PBS.

The relative permittivity of graphene $\left( {\varepsilon_{g} } \right)$ is given as^16^:

8 $$\varepsilon_{g} = 1 + \frac{{j\sigma_{g} }}{{\omega \varepsilon_{0} \Delta_{g} }}$$

where $\sigma_{g} = \sigma_{{{\text{intra}}}} + \sigma_{{{\text{inter}}}}$ is the graphene conductivity described by the Kubo formula. The absorbed power $\left( {P_{abs.} = \frac{1}{2}{\text{Re}} \left( {\sigma_{g} } \right)E^{2} \propto \frac{1}{2}\frac{{E \cdot {\text{Im}} \left( {\varepsilon_{g} } \right)}}{{\left| {\varepsilon_{g} } \right|}}} \right)$ by the graphene layer reaches maximum value when $\mu_{c} = 0.51\;{\text{eV}}$. Therefore, to control the output of the proposed PBS, the graphene layer must be used in the HSQ layer that is responsible for transferring power to the output port^16^.

The electric field distributions of controllable HPW-based PBS are demonstrated in Fig. S8b,c in the Supplementary Information. By choosing $\mu_{c} = 0\,\,{\text{eV}}$, the second mode of dielectric waveguide leads to the excitation of fundamental TM mode in the HPW. However, when $\mu_{c} = 0.51\,\,{\text{eV}}$, the excited power of HPW absorbs by the multilayer graphene and the transmission power to the HPW output port reduces significantly at 193.5 THz. In order to verify our prediction based on electric field distribution, the transmission spectra of HPW output power for two chemical potentials of 0 and 0.51 eV are plotted in Fig. S9 in the Supplementary Information.

Moreover, in Table S4 in the Supplementary Information, the suggested dynamic HPW-based PBS is compared with other graphene-based PBS.

The stacking of graphene layers within a material like HSQ can introduce challenges related to fabrication, integration, and overall device performance. Firstly, the process of stacking multiple layers of graphene inside the HSQ must be optimized to ensure precise alignment and uniformity. Any misalignment or variation in the thickness of the stacked layers can adversely impact the device performance. In addition, the compatibility between graphene and HSQ in terms of their material properties such as thermal expansion coefficients and mechanical stability should be evaluated. Mismatches in these properties can lead to mechanical stress, delamination, or other issues that can degrade the device reliability and performance over time. Furthermore, the impact of introducing multiple layers of graphene on the overall electrical and optical characteristics of the PBS needs to be carefully studied. The presence of additional graphene layers can affect the transmission properties, signal propagation, and power dissipation within the device. Therefore, it is crucial to address these practical considerations and provide insights into the fabrication techniques, characterization methods, and performance evaluation of the device when incorporating multiple layers of graphene inside the HSQ.

## HPW-based 1 × 3 power splitter

**Figure 7:**
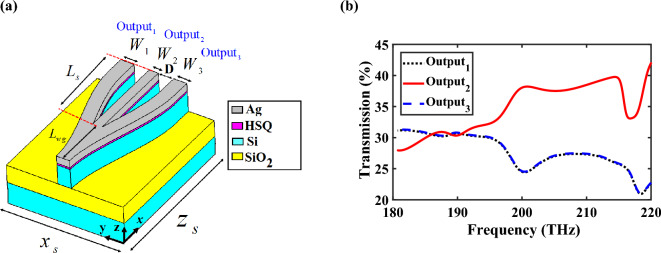
(**a**) 3D schematic view and (**b**) transmission spectra of the 1 × 3 HPW-based power splitter. The geometrical parameters are *W*_1_ = *W*_3_ = 370 nm, *W*_2_ = 186 nm, *L*_wg_ = 1230 nm, *L*_s_ = 1590 nm, *D* = 265 nm, and $x_{s} \times z_{s} = 2400 \times 2820\;{\text{nm}}^{{2}}$. Other parameters are the same as Fig. 1a.

Since the proposed CHPIC consists of different HPW-based devices, in order to redirect the input lightwave to the selected input port of each device, we need a power splitter with switchable output ports. The 3D schematic view of the designed HPW-based power splitter without switchable performance is demonstrated in Fig. 7a that is composed of a HPW waveguide divided into three asymmetric output branches. The branches are designed asymmetrically to ensure equal power distribution among the output ports.

For dividing the input power equally between three output ports, the share of each branch will be 33.33%, but due to the losses in HPW and the bend created in the lateral branches, the share of each branch will be less than that value. Examination of the transmission spectra, shown in Fig. 7b, confirms the accuracy of this prediction that the output power of the straight and lateral branches are 31.91%, 30.17%, and 30.17%, respectively, at 193.5 THz. Since the bending loss of the lateral branches are higher than the straight branch, the transmission power through the lateral branches is less than the straight one.

In the design of HPW-based power splitter, two geometric parameters of branch width and the distance between the lateral and straight branches play essential roles. The effects of these parameters are studied in section S4 of the Supplementary Information.

## Controllable HPW-based power splitter

**Figure 8:**
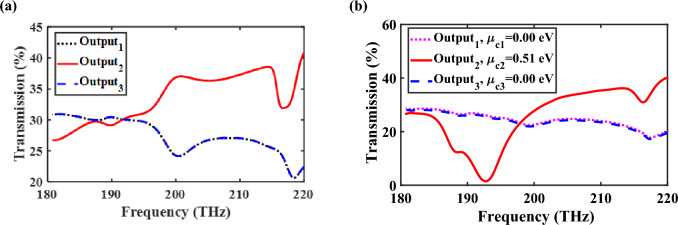
Transmission spectra of switchable power splitter when (**a**) $\mu_{c1} = \mu_{c2} = \mu_{c3} = 0\;{\text{eV}}$ and (**b**) when $\mu_{c1} = \mu_{c3} = 0\;{\text{eV}}$ and $\mu_{c2} = 0.51\;{\text{eV}}$.

In order to transmit the lightwave to the desired output of the proposed 1 × 3 power splitter and set the other ports at OFF state, eight graphene sheets are used inside the HSQ layer. Obviously, due to the variation of effective refractive index of the proposed structure by applying graphene sheets, the output power of branches changes slightly. Therefore, it is necessary to study the transmission spectra of switchable HPW-based power splitter for the chemical potential of 0 eV, as demonstrated in Fig. 8a. The output powers of straight and lateral branches are 30.52%, 29.91%, and 29.91%, respectively, at 193.5 THz. Moreover, the transmission spectra of the proposed power splitter for $\mu_{c1} = \mu_{c3} = 0\;{\text{eV}}$ and $\mu_{c2} = 0.51\;{\text{eV}}$ are plotted in Fig. 8b. The powers at the output ports of 1 to 3 are 29.87%, 0.14% and 29.87%, respectively, at the frequency of 193.5 THz. By utilizing graphene with the chemical potential of 0.51 eV at the middle branch, the output power is significantly reduced from 30.52 to 0.14%, which depicts this port is at OFF state. In lateral branches graphene acts as a transparent medium and so their output ports have considerable power. However, the graphene inside the straight branch acts as an absorbing medium and the output power at this port reaches its minimum value. Similar results are obtained by changing the chemical potential of graphene in other branches, which indicates the transmission power can be easily controlled.

The number of graphene layers is a key factor to control the output power and its results are illustrated in Table S6 in the Supplementary Information.

**Figure 9:**
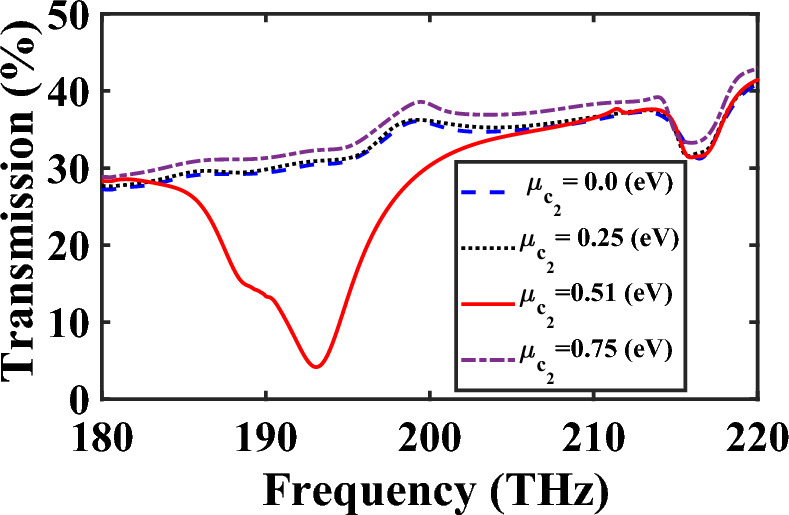
Transmission spectra of the middle branch of the power splitter for different values of $\mu_{{c_{2} }}$.

To examine the influence of chemical potential of graphene and the significant ability of $\mu_{c} = 0.51\;{\text{eV}}$ to absorb the excited TM mode, the transmission spectra of the middle branch of the power splitter are shown in Fig. 9 for different $\mu_{c2}$. The obtained results confirm that for $\mu_{c2} = 0.51\;{\text{eV}}$, it acts as an absorptive medium, while for other values it functions as a transparent medium, allowing the excited mode to transfer to the output ports.

**Figure 10:**
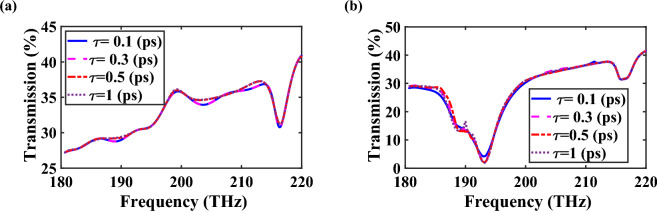
Transmission spectra of the middle branch of proposed controllable power splitter for different values of $\tau$ when (**a**) $\mu_{c2} = 0\;{\text{eV}}$ and (**b**) $\mu_{c2} = 0.51\;{\text{eV}}$.

The lifetime of plasmonic devices can be influenced by destructive processes such as conversion of plasmons into photons and electron–hole pairs through radiative processes and inter- or intra-band Landau damping, respectively^31^. However, in the case of power splitter, the plasmon excitations occur in the frequency range of 180–220 THz, which lies outside the Landau damping regime (17–53 THz)^31^. Therefore, the contribution of these damping factors is minimal. Moreover, the impact of inelastic and elastic scattering processes on graphene structures is negligible due to the insignificance of radiative damping^31^. However, to better understand the effect of scattering rate, it is crucial to examine the relaxation time of graphene on the performance of the splitter. In this regard, the transmission spectra of the power splitter of Fig. 7 are studied for two different values of chemical potential ($\mu_{c2} = 0\;{\text{eV}}$ and $\mu_{c2} = 0.51\;{\text{eV}}$). As illustrated in Fig. 10, when the relaxation time of graphene $\left( \tau \right)$ is increased from 0.1 to 1 ps, a slight enhancement in transmission power is observed. This can be attributed to the reduction in the imaginary part of the dielectric constant of graphene, leading to decreasing loss.

It is important to note that the relaxation time of graphene is influenced by the Coulomb scattering caused by charged impurities present in the substrate. This relaxation time is dependent on the Fermi energy (*E*_*F*_) and can be expressed as $\tau^{ - 1} = \frac{{4E_{F} }}{{\pi \gamma^{2} }}\int_{0}^{1} {{\text{d}}\eta \eta^{2} } \sqrt {1 - \eta^{2} } \left| {\frac{\vartheta \left( \eta \right)}{{\varepsilon \left( \eta \right)}}} \right|^{2}$, where $\hbar^{ - 1} \gamma$, $\varepsilon \left( \eta \right)$, $\vartheta \left( \eta \right)$, and $\eta$ represent the Fermi velocity, random phase approximation dielectric function, scattering potential, and momentum transfer, respectively^32^. Consequently, the optimum relaxation time varies for each device when different values of the Fermi level are selected. Overall, the effects resulting from the relaxation time are minimal and can be considered negligible.

## HPW-based coupler

**Figure 11:**
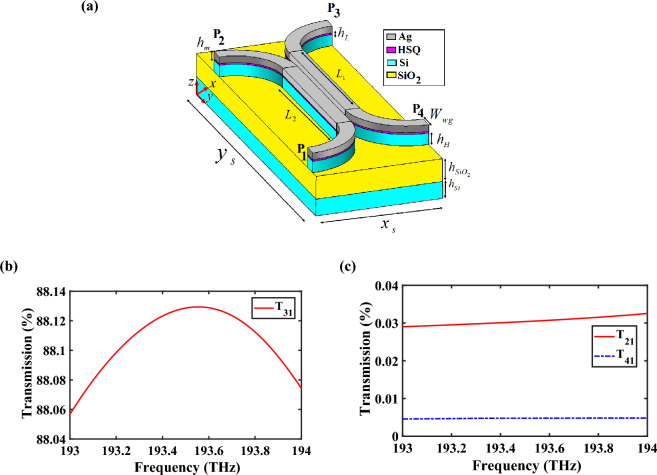
(**a**) 3D schematic view of the proposed HPW-based coupler. Transmission spectra of the (**b**) coupled port and (**c**) transmitted and isolated ports. The geometrical parameters are *L*_1_ = 3230 nm, *L*_2_ = 3080 nm, *W*_*wg*_ = 320 nm, and $x_{s} \times y_{s} = 2700 \times 6870\;{\text{nm}}^{{2}}$. Other parameters are the same as Fig. 1a.

HPW-based coupler is one of the most important devices in CHPIC that may be used for power splitting, filtering, polarization splitting, sensing, modulation, and switching^33^. It basically divides input optical signal into two output signals for a specific ratio. The 3D schematic view of the suggested HPW-based coupler is shown in Fig. 11a. P_1_ is the input port, P_2_ and P_3_ are the output transmitted and coupled ports, respectively, and P_4_ is the isolation port. Figure 11b,c demonstrates the transmission spectra of the transmitted, coupled and isolated ports. The transmission powers to the output ports of P_2_, P_3_, and P_4_ are 0.031%, 88.13% and 0.005%, respectively, at 193.5 THz. The obtained results depict that the minimum power is transferred to P_2_, and P_4_ is well isolated from other ports. By the excitation of coupler through P_1_ and determining an appropriate coupling length, the maximum power is evanescently coupled to the coupled port. Therefore, by controlling the coupling length of two branches, the input power can be coupled into the opposite branch inside the HSQ layer and transferred to the desired output. In this design, the goal is transferring the maximum power to P_3_. In Table S7 in the Supplementary Information, the effect of coupling length on the received power of all three output ports at 193.5 THz is investigated. It is obvious that the optimal response is obtained for *L*_1_ = 3230 nm and *L*_2_ = 3080 nm.

## Circular HPW-based filter, refractive index and temperature sensors

**Figure 12:**
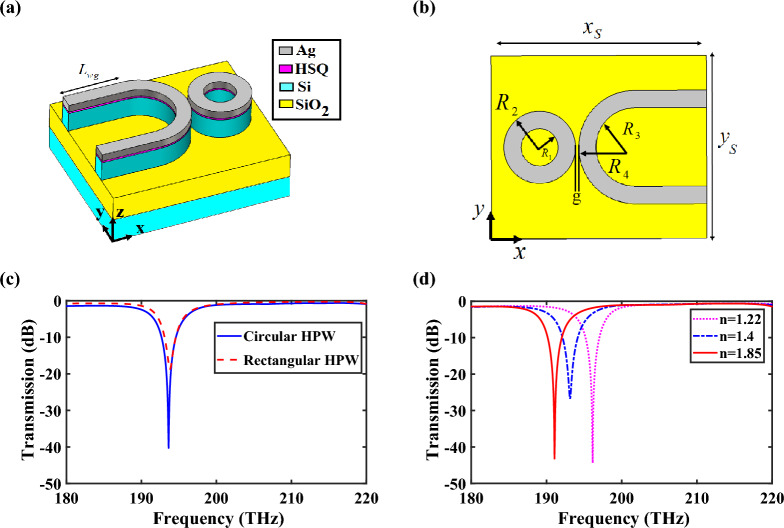
(**a**) 3D schematic and (**b**) top views of HPW-based filter with circular HPW. (**c**) Transmission spectra of the filter with rectangular and circular HPWs. (**d**) Transmission spectra of the sensor for different refractive indices of nano-fluidic channel. The geometrical parameters are *g* = 45 nm, *L*_*wg*_ = 2630 nm, *R*_1_ = 270 nm, *R*_2_ = 520 nm, *R*_3_ = 565 nm, *R*_4_ = 815 nm, and $x_{s} \times y_{s} = 3120 \times 1890\;{\text{nm}}^{{2}}$.

Ring resonators are essential component of optical and telecommunication systems and devices such as narrow- and wide-band filters, intensity or phase modulators, wavelength splitters and demultiplexers, switches, optical delay lines, and sensors^34^. Bragg-grating filters are too long due to the use of gratings. In contrast, tooth-shaped filters have smaller size, but their bandwidth is too large and not suitable for narrow-band filters. However, ring resonator-based filters show narrow bandwidth. Hence, due to the important performance of narrow-band filters as the motivation behind designing plasmonic bio and refractive index sensors, various configurations of advanced sensors have been studied due to the increasing demand for biochemical analysis and accurate detection of toxic gases or other types of materials^35^. Optical sensors based on metal–insulator-metal (MIM) waveguides have been considered by researchers due to their relatively high sensitivity, immunity to electromagnetic interference, and ease of fabrication^36^. Additionally, these sensors with different geometries have been used as a promising solution for development of PICs to measure the refractive index of materials^37^ for some applications like identifying different types of fluids and blood, measuring concentration and diagnosis of diseases^35–37^. Meanwhile, with the development of HPW technology with lower losses compared to MIM structures and high light confinement inside the layers with dimensions less than ten nanometers, it is a good choice for refractive index sensors. Therefore, in this section, the performance of HPW-based narrow-band filter and refractive index sensor have been investigated utilizing ring resonator with two different waveguides, including circular and rectangular, which are depicted in Fig. 12a,b and Fig. S12 in the Supplementary Information, respectively. The motivation for designing HPW-based filter with circular waveguide is its high efficiency compared to the rectangular one, because increasing the coupling region makes it possible to significantly increase the distance between ring resonator and waveguide, which leads to the enhancement of possibility of fabrication process. The transmission spectra of two proposed filters are illustrated in Fig. 12c that verifies the performance of filter with circular HPW is much better than the structure with rectangular HPW.

The performance of the proposed filter can be evaluated by measuring two important parameters of full-width at half-maximum (FWHM) and extinction ratio $\left( {{\text{ER}}\,\,({\text{dB}}) = \left| {10\log \left( {{{P_{out} } \mathord{\left/ {\vphantom {{P_{out} } {P_{in} }}} \right. \kern-0pt} {P_{in} }}} \right)} \right|} \right)$, where *P*_*in*_ and *P*_*out*_ are the incident and transmitted powers, respectively. The FWHM and ER of the structure with rectangular waveguide are 32 nm and 22 dB, respectively, while utilizing circular HPW reduces the FWHM drastically to 5 nm and increases the absolute value of ER to 40.5 dB. Moreover, the effect of critical structural parameters such as *R*_1_ and *g* on the transmission spectrum have been studied completely in Fig. S13 in the Supplementary Information.

Motivation behind designing HPW-based narrow-band filter is its application to propose refractive index sensor. To achieve this goal, the sensor configuration is considered similar to Fig. 12a with the exception that the HSQ spacer is replaced with a nano-fluidic channel containing sample of interest. In order to evaluate the sensing performance, two parameters of sensitivity $\left( {S_{n} = {{\Delta \lambda } \mathord{\left/ {\vphantom {{\Delta \lambda } {\Delta n}}} \right. \kern-0pt} {\Delta n}}} \right)$ and figure of merit (FOM = *S*_*n*_/FWHM) should be calculated, where $\Delta \lambda$ and $\Delta n$ are variations of the resonance wavelength and refractive index, respectively^35^. Variation of ER leads to the modification of FWHM, which will affect the sensing performance of the proposed sensor, because the FHWM is inversely related to the FOM. Therefore, in order to optimize the sensing performance, the minimum value of FWHM must be achieved, which is attained for *R*_1_ = 270 nm. In Table S8 in the Supplementary Information, the values of resonance frequency, FWHM, and ER are given for different values of *R*_1_.

Another effective parameter of a sensor is its resolution (*R* = Δλ_min_/*S*_*n*_) where Δλ_min_ is the minimum detectable wavelength of the system. Therefore, to calculate the resolution of the proposed sensor, an optical spectrum analyzer (model Ando AQ-6315A) with the resolution of Δλ_min_ = 0.5050 nm in the wavelength range of 400–1700 nm (176–750 THz) has been used^38^. Figure 12d shows the transmission spectra of the sensor for different refractive indices of nano-fluidic channel. According to the obtained results, the sensitivity, FOM and resolution of the proposed sensor are *S*_*n*_ = 133 nm/RIU, FOM = 26.6 1/RIU and *R* = 0.00375 RIU, respectively, for $\Delta \lambda = 24\,\,{\text{nm}}$ and $\Delta n = 0.18$, which leads to getting the best response.

In practical integrated circuits, the ability to monitor and control environmental parameters such as temperature is highly advantageous. Temperature variations can significantly impact the performance and reliability of integrated devices, making temperature sensor an essential component in intelligent and controllable PICs. Therefore, by incorporating temperature-sensitive materials or structures such as thermally responsive polymers or temperature-dependent resonators the proposed filter could provide dual functionality as both refractive index sensor and temperature sensor. To support this approach, in order to enhance the measurement of temperature sensitivity, we have opted for In_0.53_Al_0.1_Ga_0.37_As as an alternative to the HSQ layer. In_0.53_Al_0.1_Ga_0.37_As exhibits a temperature-dependent refractive index, making it suitable for our purpose. At temperatures below 40 K, the refractive index of In_0.53_Al_0.1_Ga_0.37_As remains relatively constant at approximately 3.462 ± 0.005. This value is 0.073 lower than the refractive index at room temperature, which is 3.535^39^. Based on the obtained experimental results, the temperature-dependent refractive index of In_0.53_Al_0.1_Ga_0.37_As is approximated as follows^39^:

9 $$\begin{aligned} n_{{\text{In}_{0.53} \text{Al}_{0.1} \text{Ga}_{0.37} \text{As}}} & = \left( {3.4615 \pm 1.1 \times 10^{ - 3} } \right) + \left( {8 \times 10^{ - 6} \pm 3.2 \times 10^{ - 5} } \right)T \\ & \quad + \left( {1.47 \times 10^{ - 6} \pm 2.5 \times 10^{ - 7} } \right)T^{2} + \left( { - 2.21 \times 10^{ - 9} \pm 5.2 \times 10^{ - 10} } \right)T^{3} \\ \end{aligned}$$

**Figure 13:**
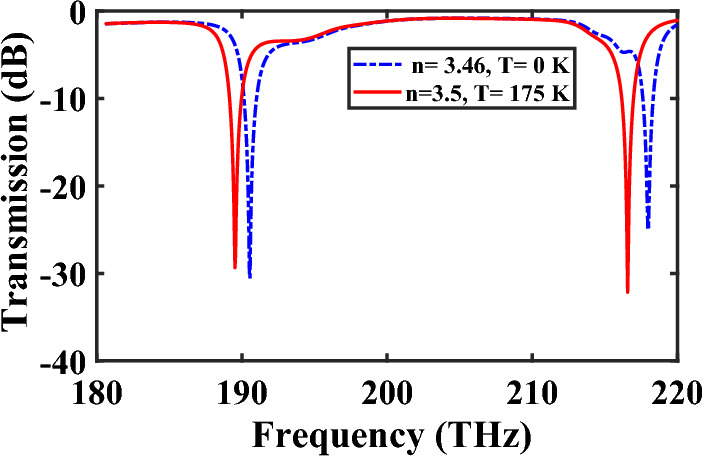
Transmission spectra of the temperature sensor at two different temperatures and their related refractive indices of In_0.53_Al_0.1_Ga_0.37_As.

To evaluate the performance of the temperature sensor, the transmission spectra at two different temperatures of 0 and 175 K are depicted in Fig. 13. The temperature sensitivity can be calculated using the formula of $S_{T} = {{\Delta \lambda } \mathord{\left/ {\vphantom {{\Delta \lambda } {\Delta T}}} \right. \kern-0pt} {\Delta T}}$, where Δ*T* represents the variation in temperature^40^. Numerical results show that the refractive index sensitivity of In_0.53_Al_0.1_Ga_0.37_As reaches 250 nm/RIU, while the temperature sensitivity reaches 0.05 nm/K. As a result, by incorporating a temperature sensor in addition to the refractive index sensor, the proposed filter could offer enhanced functionality and broaden its applications in practical integrated circuits.

## Controllable hybrid plasmonic integrated circuit

**Figure 14:**
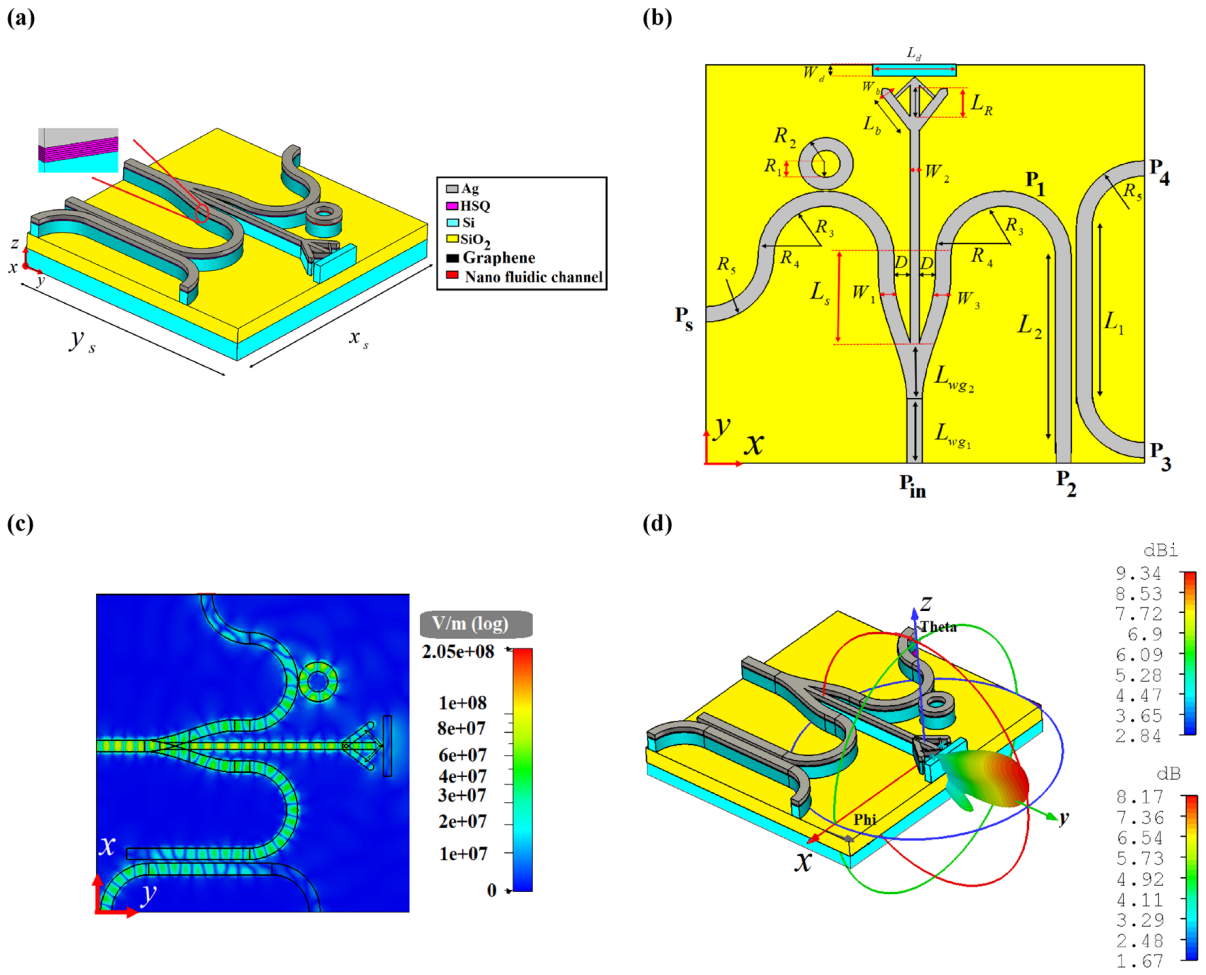
(**a**) 3D and (**b**) 2D schematic views of the CHPIC with four components of nano-antenna, power splitter, coupler and sensor. (**c**) Electric field distribution of the proposed CHPIC at the center of HSQ layer and (**d**) 3D radiation pattern of the nano-antenna at 193.5 THz. Geometrical parameters are *L*_1_ = 3080 nm, *L*_2_ = 3230 nm, *L*_*R*_ = 829 nm, *L*_*b*_ = 915 nm, *L*_*s*_ = 1590 nm, *L*_*d*_ = 1600 nm,* L*_*wg*1_ = 1290 nm,* L*_*wg*2_ = 1230 nm,* W*_1_ = 370 nm,* W*_2_ = 186 nm,* W*_3_ = 370 nm, *W*_*d*_ = 225 nm, *W*_*b*_ = 170 nm, *R*_1_ = 270 nm, *R*_2_ = 520 nm,* R*_3_ = 565 nm,* R*_4_ = 815 nm, *R*_5_ = 150 nm, *D* = 265 nm, *x*_*s*_ × *y*_*s*_ = 920 × 8386 nm^2^.

Integrated circuits are indispensable in communication systems, as they lower the cost of system by making a compact circuitry unlike discrete components. The most important aim to investigate different hybrid plasmonic devices in previous sections is to propose a CHPIC. It is essential to mention that the performances of the proposed hybrid plasmonic components have been optimized based on their functionality in the investigated CHPIC. As depicted in Fig. 14a,b, the suggested CHPIC is comprised of four major components of 1 × 3 dynamic power splitter, sensor, transmitter nano-antenna, and coupler. To design CHPIC with the capability of controlling the output power, a multilayer graphene inside the HSQ layer of the output branches of the power splitter has been used. According to our observations, if the chemical potential of graphene inside each branch is considered 0.51 eV, due to the high absorption feature of graphene, quite low power will be transferred to the selected output. In other words, based on the unique property of graphene, the output of the power splitter can be turned ON and OFF. Therefore, the performance of each component connected to the output branches of the power splitter can be quickly controlled. For the initial evaluation of the proposed chip functionality, the chemical potential of graphene in each branch is 0 eV.

The CHPIC demonstrated in Fig. 14a is excited by TM mode through the HPW and the power is divided to the branches of the power splitter connected to other devices. In other words, the nano-antenna, sensor and coupler can be stimulated through the output power of the power splitter, as illustrated in the electric field distribution of Fig. 14c. The 3D radiation pattern of the nano-antenna, which is connected to the middle branch of the power splitter is shown in Fig. 14d with the gain and directivity of 8.17 dB and 9.34 dBi, respectively, at 193.5.

**Figure 15:**
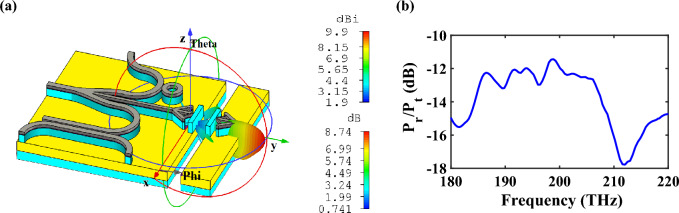
(**a**) 3D radiation pattern and (**b**) the spectrum of the ratio of the received power (*P*_*r*_) to the transmitted power (*P*_*t*_) of the wireless transmission link.

It can be seen that the nano-antenna has an optimized performance with horizontal radiation pattern that is suitable for proposing an on-chip wireless transmission link, as demonstrated in Fig. 15a. By placing a receiver nano-antenna in another chip, the information can be transferred from the CHPIC to another chip. In this way, without increasing the complexity, it is possible to establish communication between two chips in different layers of the system and transfer data from one chip to another one with the maximum gain and directivity of 8.74 dB and 9.9 dBi, respectively, at 193.5 THz. Figure 15b depicts the spectrum of the received power to the transmitted power ratio (*P*_*r*_/*P*_*t*_) for the distance of *d*_link_ = 700 nm between two different chips. The obtained value of *P*_*r*_/*P*_*t*_ at 193.5 THz is − 12.05 dB, which verifies that by utilizing the optimized HPW-based nano-antenna in the CHPIC to design an on-chip wireless transmission link, the system will not experience much propagation loss. It is because by squeezing the light into the layer with low refractive index (HSQ layer), which its thickness is typically much smaller than the applied wavelength, the CHPIC idea can obstacle the inherent loss issue of plasmonic materials at optical frequencies, which has restricted further applications of plasmonic devices. The propagation loss due to the metal absorption in HPWs is in the order of 0.01 dB/μm, which is much lower than traditional ones^41^.

**Figure 16:**
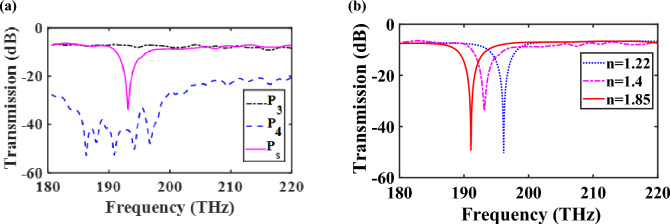
(**a**) Transmission spectra of (**a**) the CHPIC from the output ports of the coupler and sensor and (**b**) the sensor embedded in the CHPIC for different refractive indices of the nano-fluidic channel.

Studying the transmission power to the coupler and sensor in the CHPIC determines that the guided TM mode to the lateral branches of the power splitter leads to the excitation of both devices. As shown in Fig. 16a, the power transmitted to the output ports of P_3_ (coupled port), P_4_ (isolated port) and P_s_ (sensor output port) are − 7.319, − 43.2, and − 31.7 dB, respectively, at 193.5 THz, which represents the coupled and isolated arms of the coupler have optimized performance because the significant amount of the power is coupled into P_3_. Our investigation reveals that transmitted and isolated arms (P_2_ and P_4_ ports) introduce some cross talk, which results in deteriorated output signal. Hence, in order to ignore this effect, it is better to optimize the interaction coupler length. The track length of the S-shaped bend of lateral branches should be computed using the equation of $\int\nolimits_{0}^{{\min (L_{1} \& L_{2} )}} {\left( {\sqrt {\left( {f^{\prime}\left( t \right)} \right)^{2} + 1} } \right)} \,dt$, where $f\left( t \right)$ is the S-bend function equation^42^. The coupled power reduces on increasing the coupler length further due to the increased ohmic losses, as the total track length of the bend of HPW-based power splitter increases. Also, the obtained FWHM of left lateral branch coupled to the ring resonator is 9 nm, which is increased 4 nm compared to the attained FWHM of the HPW-based sensor examined separately in the previous section due to increasing the ohmic loss. This effect leads to the reduction of FOM of the embedded sensor in the proposed CHPIC.

As it is mentioned before, the combination of guiding in low-index medium (HSQ layer) and polarization diversity of HPWs can be very beneficial for sensing. The proposed sensor consists of a silver surface separated from a high-index region (silicon or silicon nitride) by a nano-fluidic channel. As shown in Fig. 14c, for the TM mode, a significant amount of power is concentrated in the nano-fluidic channel. Although, the TE mode is concentrated in silicon layer, part of the evanescent mode will also be in the nano-fluidic channel of HPW-based sensor. Therefore, the TE mode supported by HPW can also be used for sensing. As a result, the polarization diversity of HPW can be used for overcoming the limitations of plasmonic sensors^43^ in the following ways:

1. Because of polarization diversity, the HPW-based sensor can be exploited to find additional structural information about complex molecules, similar to dual polarization interferometer.
2. Since TM mode is tightly confined into the nano-fluidic channel of the sensor, higher sensitivity can be obtained compared to the plasmonic one. Based on this attitude and the obtained results extracted from Fig. 16b, the sensitivity, FOM and resolution of the sensor are *S* = 133 nm/RIU, FOM = 14.77 1/RIU and R = 0.00375 RIU, respectively, for $\Delta$n_nano-fluidic_ = 0.18 and $\Delta \lambda = 24\;{\text{nm}}$.
3. Based on the similar idea proposed in^44^, the contributions from bulk $\left( {\frac{{\partial n^{\prime}_{eff} }}{{\partial n_{c} }} = \frac{{n^{\prime}_{eff} \left( {n_{c} + \Delta n_{c} } \right) - n^{\prime}_{eff} \left( {n_{c} - \Delta n_{c} } \right)}}{{2\Delta n_{c} }}} \right)$ and surface sensitivities $\left( {\frac{{\partial n^{\prime}_{eff} }}{\partial a} = \frac{{n^{\prime}_{eff} \left( {a + \Delta a} \right) - n^{\prime}_{eff} \left( {a - \Delta a} \right)}}{2a}} \right)$ can be separated to determine non-zero sensitivity matrix (*S*) as follows:
  10 $$S = \left( {\begin{array}{*{20}c} {\frac{{\partial n^{\prime}_{eff} }}{{\partial n_{c} }}\left( {TE} \right)} & {\frac{{\partial n^{\prime}_{eff} }}{\partial a}\left( {TM} \right)} \\ {\frac{{\partial n^{\prime}_{eff} }}{{\partial n_{c} }}\left( {TM} \right)} & {\frac{{\partial n^{\prime}_{eff} }}{\partial a}\left( {TE} \right)} \\ \end{array} } \right)$$
  11 $$\Delta n_{eff} \left( {TE} \right) = \frac{{\partial n^{\prime}_{eff} }}{{\partial n_{c} }}\left( {TE} \right)\Delta n_{c} + \frac{{\partial n^{\prime}_{eff} }}{\partial a}\left( {TE} \right)\Delta a$$
  12 $$\Delta n_{eff} \left( {TM} \right) = \frac{{\partial n^{\prime}_{eff} }}{{\partial n_{c} }}\left( {TM} \right)\Delta n_{c} + \frac{{\partial n^{\prime}_{eff} }}{\partial a}\left( {TM} \right)\Delta a$$

where *n*_*c*_, $\Delta n_{c}$, *a* and $\Delta a$ are refractive index and variation of bulk index of the fluid, adlayer thickness and variation of adlayer thickness, respectively, which are related to the changes of effective index of fundamental TE $\left( {\Delta n_{eff} \left( {TE} \right)} \right)$ and TM $\left( {\Delta n_{eff} \left( {TM} \right)} \right)$ modes that are known from measurements. Also, $\Delta n_{c}$ and $\Delta a$ can be determined from Eqs. (11) and (12). With this attitude, to investigate the performance of HPW-based sensor embedded in the proposed CHPIC, similar to the results of ref.^45^, we assumed *a* = 3 nm, $\Delta a$ = 0.1 nm, *n*_*c*_ = 1.4, and $\Delta n_{c}$ = 0.18. Therefore, the obtained non-zero TM elements sensitivity matrix are S_12_ = 0.000023475 and S_21_ = 3.6207.

Another important feature of the proposed CHPIC is its capability to control the transmission power by utilizing switchable HPW-based power splitter. In this regard, the absorption property of multilayer graphene is controlled through its chemical potential to govern the output power of 1 × 3 power splitter. Moreover, one approach to bias the multilayer graphene is utilizing the electrodes of Ag and platinum (P_t_) with the thicknesses of 100 and 10 nm, respectively. The distance between the graphene slot and electrodes is 420 nm because of controlling the effect of metal contact disturbance on the HPW performance^46^.

**Figure 17:**
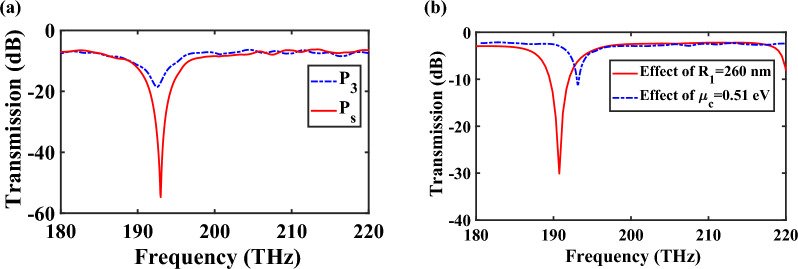
(**a**) Transmission spectra of the sensor and coupler embedded in the CHPIC when $\mu_{{c_{1} }} = \mu_{{c_{3} }} = 0.51\;{\text{eV}}$. (**b**) Transmission spectra of the proposed CHPIC for *R*_1_ = 260 nm and $\mu_{{c_{3} }} = 0.51\;{\text{eV}}$.

As shown in Fig. 17a, by applying the chemical potential of 0.51 eV for lateral branches of the power splitter $\left( {\mu_{{c_{1} }} = \mu_{{c_{3} }} = 0.51{\text{ eV}}} \right)$, the transmission spectra of HPW-based coupler and sensor embedded in the CHPIC reveal that not only the ER of HPW-based sensor (left lateral branch) is increased from 31.7 to 54.75 dB, which leads to the reduction of FWHM from 9 to 5 nm to get higher FOM, but also the power transmitted to the right lateral branch (input port of the coupler) at 193.5 THz cannot couple to the output port P_3_. As it is mentioned before, the resonance frequency can be controlled by changing the ring resonator radius, so two resonance frequencies can be simultaneously filtered by turning on the graphene in the left lateral branch connected to the sensor, without increasing the sensor design complexity. According to Fig. 17b, by choosing *R*_1_ = 260 nm and $\mu_{{c_{3} }} = 0.51{\text{ eV}}$, two resonance frequencies of 193.5 and 191 THz are achieved at the same time.

**Figure 18:**
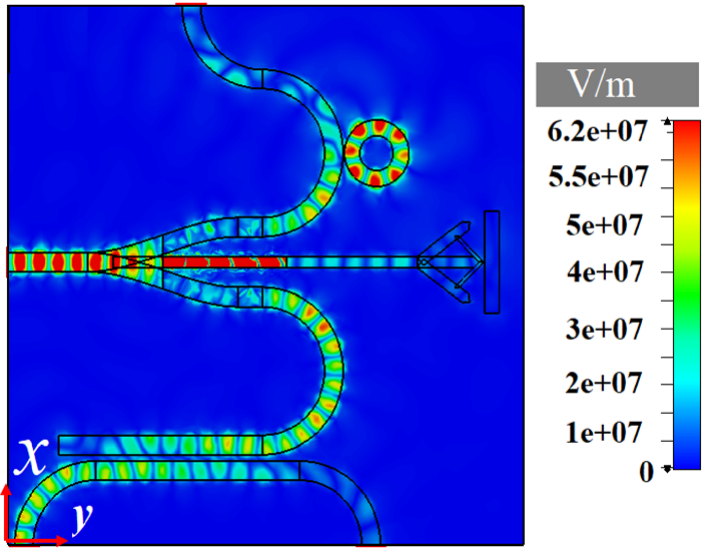
Electric field distribution of the proposed CHPIC for $\mu_{c2} = 0.51\;{\text{eV}}$.

In addition, according to the electric field distribution of the CHPIC depicted in Fig. 18, by choosing $\mu_{{c_{2} }} = 0.51\;{\text{eV}}$ for middle straight branch connected to the nano-antenna, a major amount of the transmission power is absorbed by graphene layers, leading to a sharp reduction of nano-antenna efficiency. In this case, the obtained gain and directivity are 2.41 dB and 5.03 dBi, respectively, at 193.5 THz, which is decreased in comparison to the isolated nano-antenna. It is important to note that by increasing the number of graphene layers, the power transmitted to the nano-antenna can be reduced drastically.

**Figure 19:**
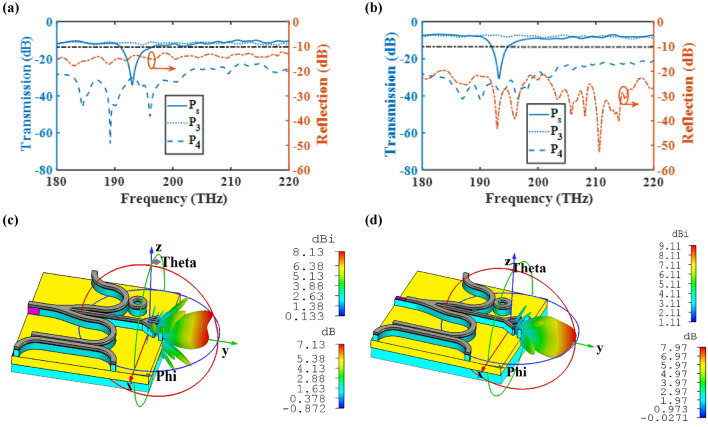
Transmission and reflection spectra and radiation patterns of the proposed CHPIC at 193.5 THz excited by (**a**,**c**) plasmonic and (**b**,**d**) dielectric waveguides.

Since the stimulation of the CHPIC may not be done directly by the laser (input port (P_in_)) and the information is transferred to the input of the switchable power splitter through the plasmonic or photonic (dielectric) waveguide, it is essential to investigate the performance of CHPIC when it is connected to another chip with different configuration. As mentioned before, the coupling efficiency of plasmonic and photonic waveguides to the HPW are 85% and 94%, respectively, at 193.5 THz. It is expected that the gain and directivity of nano-antenna change slightly due to the reduction of coupled power to the HPW. However, no significant changes will be observed for other components of CHPIC. Figure 19a,b shows the transmission and reflection spectra of the CHPIC fed by plasmonic and dielectric waveguides, respectively. The reflection spectra of both structures disclose that better impedance matching occurs when the chip is connected to the dielectric waveguide because photonic chips does not have major effect on increasing the connection loss. As a result, the radiation characteristics of the nano-antenna are more optimized compared to the case when the CHPIC is connected to the plasmonic chip. Also, the transmission spectra of HPW-based sensor and coupler prove that the connection of plasmonic and photonic chips to the CHPIC does not have a significant impact on their performance.

According to Fig. 19c,d, the radiation patterns of the CHPIC at 193.5 THz reveal that the plasmonic connection has greater effect on the nano-antenna radiation due to its higher ohmic losses and lower coupling efficiency in comparison to the dielectric one. As seen in Fig. 19c, the excitation of CHPIC through the plasmonic waveguide not only reduces the gain and directivity of the embedded nano-antenna, but also it leads to the generation of side lobes, which is not suitable for on-chip point-to-point wireless link. However, it is possible to optimize the nano-antenna performance by changing its geometrical parameters when the CHPIC is connected to the plasmonic chip. In contrast, the radiation pattern of the CHPIC connected to the dielectric chip in Fig. 19d shows that this layout has better radiation performance. Although, for both configurations we are facing with the decrease of nano-antenna gain and directivity, because of the reduction of coupling power.

**Figure 20:**
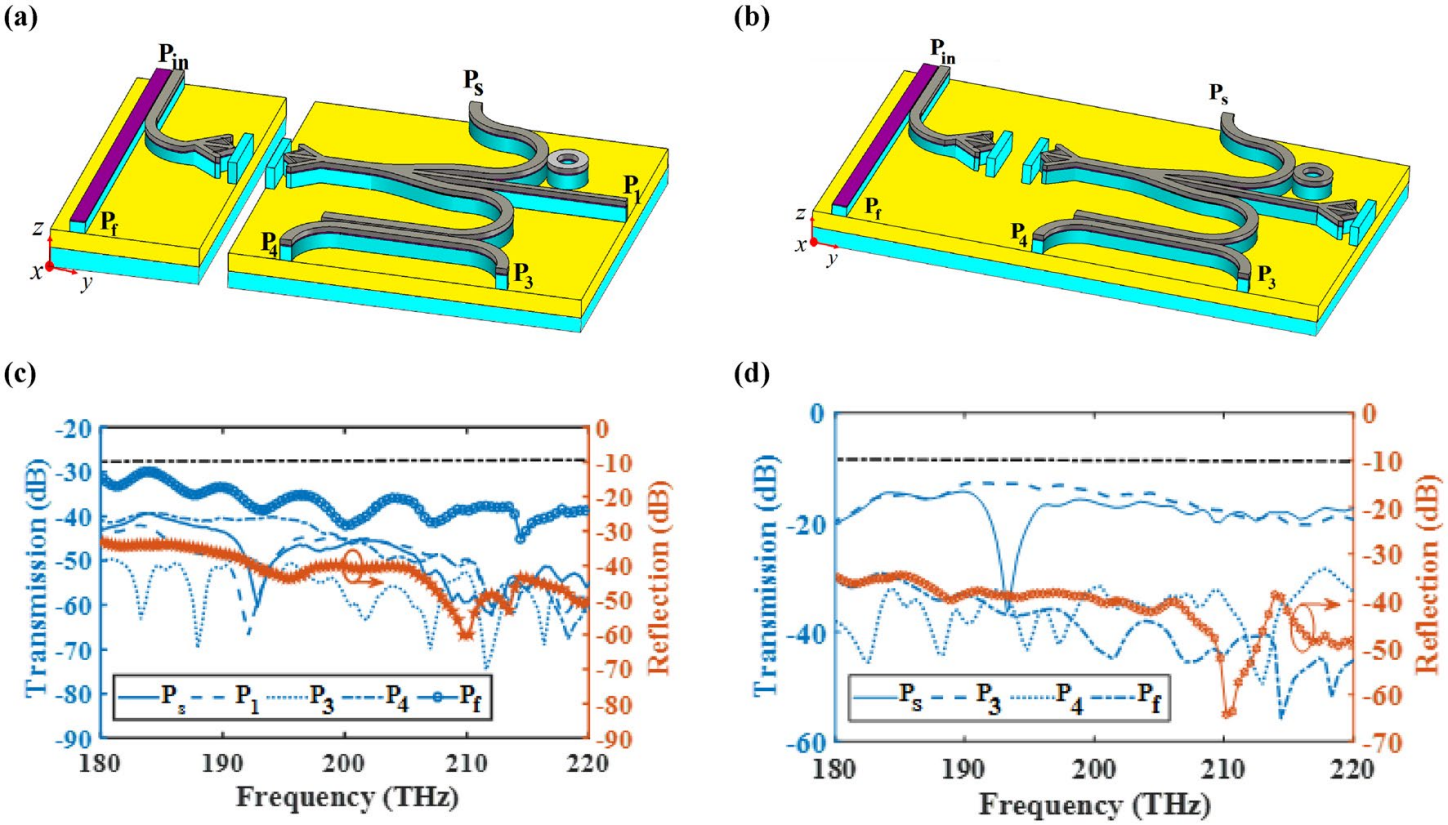
3D schematic view of proposed CHPIC connected to the wireless transmission link and its transmission spectra for output ports. (**a**,**c**) Intra and (**b**,**d**) inter on-chip excited by PBS that is connected to the transmitter nano-antenna.

**Figure 21:**
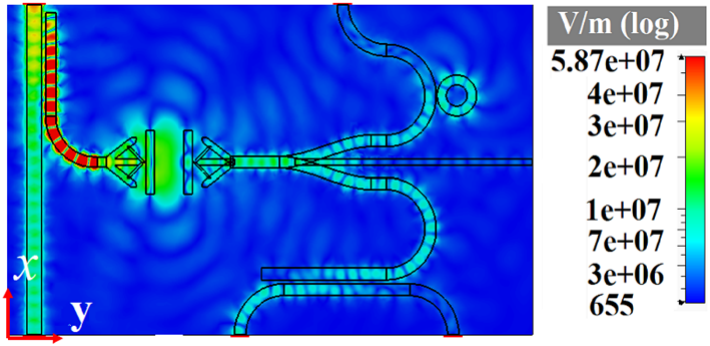
Electric field distribution of the intra on-chip wireless CHPIC at 193.5 THz when $\mu_{c2} = 0.51\;{\text{eV}}$.

Finally, the ultimate goal is to connect the PBS to the proposed CHPIC. Up to this phase, the proposed CHPIC was able to transmit chip information to another chip through the transmitter nano-antenna based on the idea of wireless on-chip link. Now, we want to propose an attractive and novel approach to excite the CHPIC through the receiver nano-antenna, which is realized by inter/intra on-chip wireless link. Motivation behind this approach is realization of controllable PIC that can get and transfer the information through the intra/inter on-chip wireless link. As depicted in Fig. 20a,b, the transmitter nano-antenna is connected to the HPW-based PBS based on two different concepts of designing intra and inter on-chip wireless connections. The transmission spectra of intra on-chip wireless CHPIC by assuming that the straight branch of power splitter is at OFF state $\left( {\mu_{c2} = 0.51\;{\text{eV}}} \right)$ are demonstrated in Fig. 20c. It is obvious that the graphene in the middle branch of the power splitter absorbs significant excited power at 193.5 THz. Therefore, the amount of transmission power from the power splitter into the output port P_1_ decreases about − 38.4 dB at 193.5 THz. In other words, this desired optical frequency is filtered at the output port of the middle branch. Also, the reflection spectrum illustrates a well*-*matched and balanced impedance matching in the proposed CHPIC and its bandwidth with the intra on-chip wireless transmission link is 40 THz. Exploring the transmission spectrum of the HPW-based PSB shows that only less than − 35 dB (3%) of the power transmits into the output port of P_f_, and more than 97% of the coupled power transfers into the HPW that excites the nano-antenna. It is important to mention that the excitation branch of the HPW-based PSB is stimulated by the TE mode and the coupled mode in the parallel HPW-based branch is TM mode, which is concentrated in the layer with the low refractive index to excite the CHPIC. The electric filed distribution of the structure of Fig. 20a at 193.5 THz is shown in Fig. 21.

Figure 20d displays the transmission spectra of the proposed CHPIC consisting of inter on-chip wireless transmission link. In the proposed CHPIC shown in Fig. 20b, the middle branch of power splitter is connected to the transmitter nano-antenna in order to transfer data from the intra on-chip wireless link to various layers of the optical circuit. The gain and directivity of the transmitter nano-antenna are 5.69 dB and 6.25 dBi, respectively, at 193.5 THz. By comparing the transmission spectra of the sensor and coupler based on Fig. 20c,d, their functionalities have not been changed by utilizing the idea of proposing the intra on-chip wireless link.

## Conclusion

In summary, different configurations of CHPIC with the capability to realize intra/inter on-chip wireless transmission link at the telecommunication frequency of 193.5 THz have been proposed, which are composed of CMOS compatible HPW-based indispensable components of nano-antenna, power splitter, sensor, filter, and polarization beam splitter. The switchable power splitter has been achieved utilizing the multilayer graphene based on amazing features of graphene as an epsilon-near-zero and absorptive/transparent material. The characteristics of each isolated proposed device have been investigated completely based on the FEM. HPW-based components are very promising for on-chip applications, as they work proficiently while they are integrated in a single chip. This study may inspire future nanoscale on-chip device, which is compatible with EBL and lift-off fabrication techniques.

## Supplementary Information

Supplementary Information.

## Data Availability

The datasets used and/or analyzed during the current study available from the corresponding author on reasonable request.
