# Supplementary material for: Controllable hybrid plasmonic integrated circuit
Source: Sci Rep. 2023 Jun 20;13:9983. doi: 10.1038/s41598-023-37228-4 (PMC10282038; doi:10.1038/s41598-023-37228-4)
Supplement: Supplementary file 1 — Supplementary Information. [file 41598_2023_37228_MOESM1_ESM.docx]

**Supplementary Information**

**Controllable hybrid plasmonic integrated circuit**

**Maryam Khodadadi1, Seyyed Mohammad Mehdi Moshiri1, Najmeh Nozhat1,*, and Mohsen Khalily2**

1 Department of Electrical Engineering, Shiraz University of Technology, Shiraz, Iran.

2 5G & 6G Innovative Centers (5GIC & 6GIC), Institute for Communication Systems (ICS), University of Surrey, Guildford, UK

*nozhat@sutech.ac.ir

**S1. Hybrid plasmonic waveguide**

The coupled mode theory (CMT) can make a physical insight to design HPW, but it is not accurate because the loss is not considered in the CMT. Therefore, for analysis of the modes supported by the HPW, the FEM and transfer matrix theory are used.

If the propagation is considered along the z direction, the magnetic ﬁeld for the intermediate layers of Si and HSQ (*m*=1, 2) can be written as:

$\text{H}_{m}\left(y,z,t\right)=\hat{x}\left[H_{m}\cos\left(k_{m}y\right)+H'_{m}\sin\left(k_{m}y\right)\right]\mathrm{e}^{-\gamma z+j\omega t}$ (S1)

where $H_{m}$ and $H'_{m}$ are constant amplitudes, $\gamma=k_{0}\left(n_{eff}+jk_{eff}\right)$ is the complex propagation constant, $k_{0}=2\pi/\lambda_{0}$ is the free space wave number, $\lambda_{0}$ is the free space wavelength, $n_{eff}$ and $k_{eff}$ are the real and imaginary parts of the effective refractive index, respectively. Also, $k_{m}=\sqrt{\varepsilon_{m}k_{0}^{2}-\gamma^{2}}$ is the transverse wave number for intermediate layers (*m*=1 for Si and *m*=2 for HSQ). Furthermore, the magnetic field in the Ag and SiO2 layers can be defined as:

$\text{H}_{Ag}\left(y,z,t\right)=\hat{x}H_{Ag}e^{-k_{Ag}\left(y-\left(h_{L}+h_{H}\right)\right)}\mathrm{e}^{-\gamma z+j\omega t}$ (S2)

$\text{H}_{SiO_{2}}\left(y,z,t\right)=\hat{x}H_{SiO_{2}}e^{k_{SiO_{2}}y}\mathrm{e}^{-\gamma z+j\omega t}$ (S3)

where $k_{Ag}=\sqrt{\gamma^{2}-\varepsilon_{Ag}k_{0}^{2}}$ and $k_{SiO_{2}}=\sqrt{\gamma^{2}-\varepsilon_{SiO_{2}}k_{0}^{2}}$ are the attenuation coefficients in the Ag and SiO2 layers, respectively. Also, to simplify the calculation of the dispersion relation, we set the zero of the coordinate system on the SiO2 layer. By using the boundary condition and finding the relation between the constant amplitudes of the substrate (SiO2) and cladding (Ag) layers, the dispersion relation for the multilayer structure can be obtained as:

$\frac{k_{SiO_{2}}}{\varepsilon_{SiO_{2}}}a_{11}+\frac{k_{Ag}}{\varepsilon_{Ag}}a_{22}-a_{21}+\frac{k_{Ag}k_{SiO_{2}}}{\varepsilon_{Ag}\varepsilon_{SiO_{2}}}a_{12}=0$ (S4)

The coefficients of *a*ij (i=1, 2; j=1, 2) are given by:

$a_{11}=\cos\left(k_{1}h_{H}\right)\cos\left(k_{2}h_{L}\right)-\frac{\varepsilon_{Si}k_{2}}{k_{1}\varepsilon_{HSQ}}\sin\left(k_{1}h_{H}\right)\sin\left(k_{2}h_{L}\right)$ (S5a)

$a_{12}=\frac{\varepsilon_{HSQ}}{k_{2}}\cos\left(k_{1}h_{H}\right)\sin\left(k_{2}h_{L}\right)+\frac{\varepsilon_{Si}}{k_{1}}\sin\left(k_{1}h_{H}\right)\cos\left(k_{2}h_{L}\right)$ (S5b)

$a_{21}=\frac{k_{1}}{\varepsilon_{Si}}\sin\left(k_{1}h_{H}\right)\cos\left(k_{2}h_{L}\right)+\frac{k_{2}}{\varepsilon_{HSQ}}\cos\left(k_{1}h_{H}\right)\sin\left(k_{2}h_{L}\right)$ (S5c)

$a_{22}=-\frac{\varepsilon_{HSQ}}{k_{2}}\cos\left(k_{1}h_{H}\right)\sin\left(k_{2}h_{L}\right)+\cos\left(k_{1}h_{H}\right)\cos\left(k_{2}h_{L}\right)$ (S5d)

To simplify the calculations, the dimension of the HPW is considered infinite along the x direction.

Figure S1 depicts the spectra of SPPs propagation length $\left(L_{p}=\lambda/4\pi n''_{eff}\right)$ and real part of the effective refractive index $\left(n'_{eff}\right)$ of HPW, where λ and $n''_{eff}$ are the target wavelength and the imaginary part of the effective refractive index, respectively. The obtained effective mode index $\left(A_{eff}=\frac{\iint W(x,y) dxdy}{\max\left\{W(x,y)\right\}}\right)$ and figure of merit $\left(\text{FOM}=L_{P}/\sqrt{A_{eff}/\pi}\right)$ of the proposed HPW are $8.4112\times10^{-14}$ and $5.8692\times10^{8}$, respectively, where $W(x,y)$ is the energy density per unit length flowed along the direction of propagationS1. These results confirm that the HPW has better performance in comparison to the similar plasmonic waveguides.

**Figure S1.** Spectra of the real part of the effective refractive index and SPPs propagation length of the proposed HPW.

**S2. Hybrid plasmonic rhombic nano-antenna**

**S2.1. Effect of coupling between HPW and plasmonic or dielectric waveguides on the HPRNA performance**

Figure S2 shows the 3D schematic views of dielectric waveguides with single silicon layer and bi-layer of silicon and HSQ, which are connected to the HPW-feed line of HPRNA. The return loss spectra of both structures are depicted in Fig. S3. As the connection of dielectric waveguide to the HPW-feed line does not involve the intrinsic losses to the HPRNA, the obtained bandwidth and impedance matching of nano-antenna do not meet major and fundamental changes. Also, the gain and directivity spectra of Fig. S3 disclose that the far-field features of HPRNA are improved for both cases. The gain and directivity for single silicon layer are 11 dB and 11.2 dBi, respectively, and for bi-layer of silicon and HSQ are 11.8 dB and 12 dBi, respectively, at 193.5 THz. Therefore, the performance of bi-layer dielectric waveguide is better than silicon waveguide to enhance the far‑field characteristics of the HPRNA.

| **(a)** | **(b)** |
| --- | --- |
| 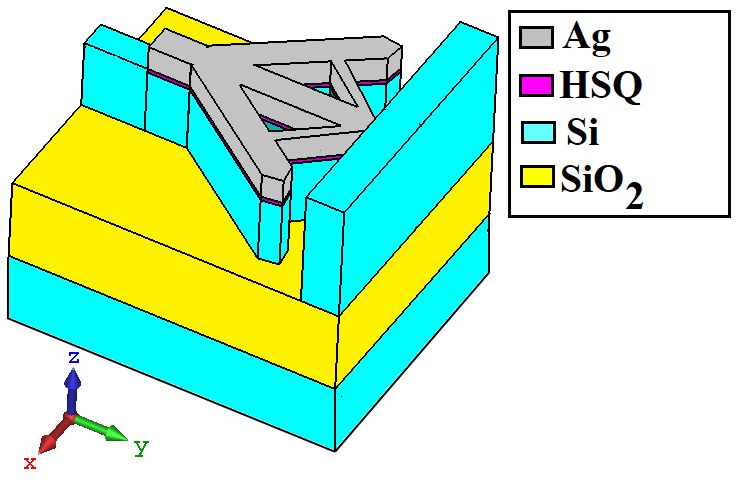 | 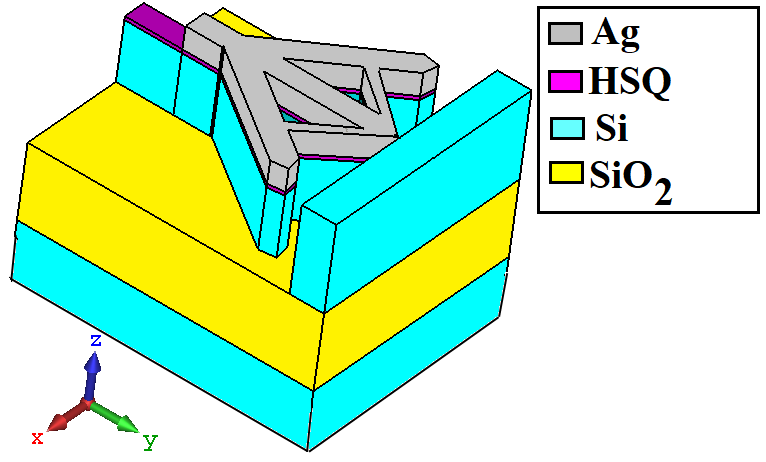 |
| **Figure S2.** 3D schematic views of dielectric waveguide connection to the HPW with (a) single silicone layer and (b) bi-layer dielectric guiding consists of two layers of silicon and HSQ. | |

| **(a)** | **(b)** |
| --- | --- |
|  |  |
| **Figure S3.** Reflection coefficient, gain and directivity spectra of the HPRNA connected to (a) the single silicon layer and (b) bi-layer of silicon and HSQ. | |

The following equation is used to calculate the coupling efficiency when the HPW-feed line is connected to the plasmonic and dielectric waveguidesS2:

Coupling Efficiency (dB) = (1- | S11|2) × (radiation efficiency) (S6)

By extracting data from Fig. S3 and using Equation (S6), the coupling efficiency spectra of Fig. S4 are obtained that illustrate the coupled power is more than 90% and 85% for single and bi-layer dielectric connections to the HPW-feed line, respectively, in the desired frequency range.

| **(a)** | **(b)** |
| --- | --- |
|  |  |
| **Figure S4.** Coupling efficiency spectra of HPRNA connected to the (a) single silicon layer and (b) bi‑layer of silicon and HSQ. | |

Another challenge that needs to be considered is the connection of plasmonic waveguide to the HPW, depicted in Fig. S5a, and its effect on the nano-antenna far-field performance, bandwidth and coupling efficiency. The reflection coefficient of Fig. S5b demonstrates that this concept leads to the impedance mismatch at many frequencies, and reducing the nano-antenna bandwidth because of increasing the intrinsic losses, which plasmonic waveguide applies to the whole structure. Therefore, compared to the dielectric waveguide connection, utilizing plasmonic waveguide has a significant effect on the impedance matching and bandwidth. In addition, because of the intrinsic loss of plasmonic waveguide, the nano‑antenna efficiency reduces from 95.5% to 78.21% at 193.5 THz and the obtained gain reaches 9.67 dB. As shown in Fig. S5c, the coupling efficiency of plasmonic waveguide connection to the HPW is decreased in comparison to dielectric one, which has a major effect on the HPRNA efficiency because less transmission power converts to radiation power and propagates to the free space.

In the following, we will examine the importance of feeding the flared radiation part with the proposed HPW instead of plasmonic waveguide-feed line to improve the HPRNA performance. Obviously, plasmonic waveguide‑feed line is not a suitable candidate because of its higher propagation loss compared to the HPW-feed line, which leads to the reduction of nano-antenna gain and efficiency. As illustrated in the 3D directivity radiation pattern of HPRNA with plasmonic waveguide-feed line in Fig. S5d, not only the nano-antenna gain decreases from 10 dB to 9.67 dB, but also it leads to the displacement of the main lobe towards the substrate which impairs the performance of HPW-based components in the proposed CHPIC. As a result, by utilizing the idea of ​​HPW, the characteristics of HPRNA are significantly improved compared to the corresponding plasmonic waveguide-feed nano‑antennas. It should be noted that if Si is used instead of HSQ for the design of plasmonic waveguide-feed line, the propagation length, reflection coefficient and far-field performance of HPRNA will be much worse.

| **(a)** | **(b)** | |
| --- | --- | --- |
| 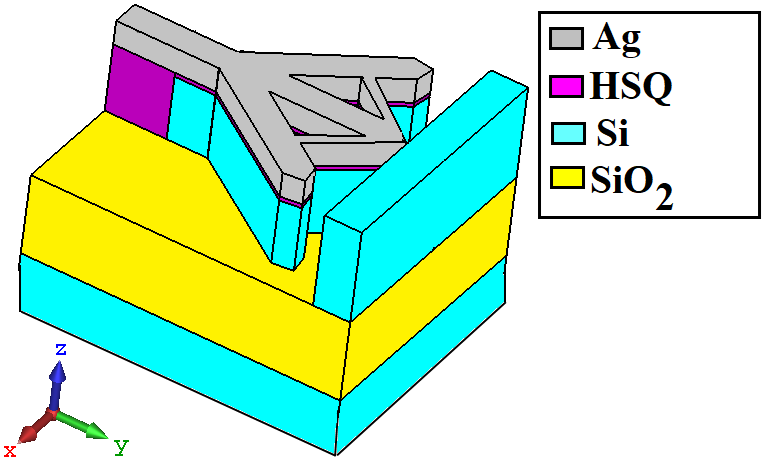 |  | |
| **(c)** | **(d)** | |
|  | 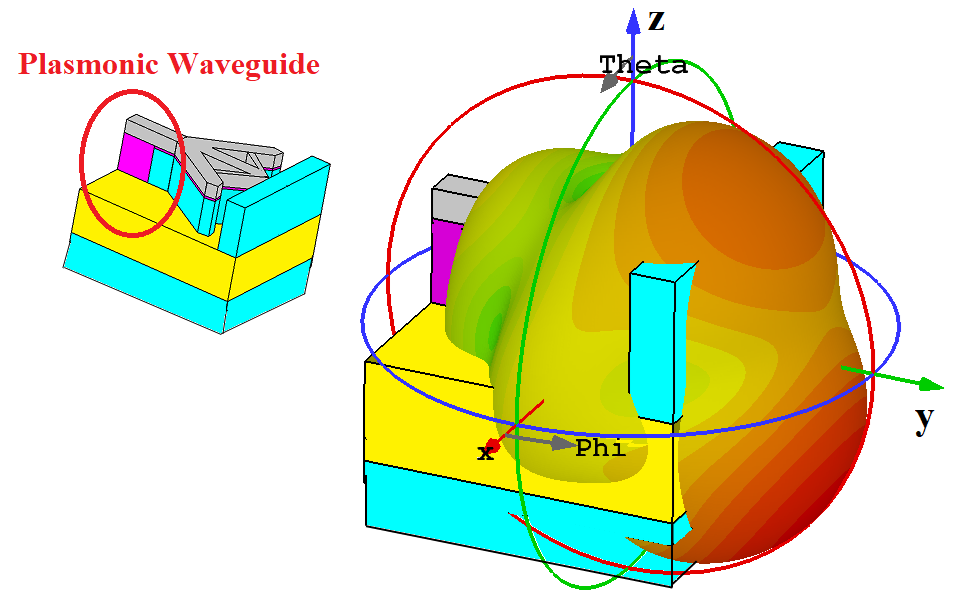 | 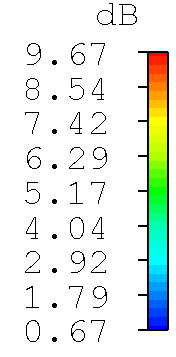 |
| **Figure S5.** (a) 3D schematic view and (b) reflection coefficient, gain, and directivity spectra of the HPRNA with plasmonic waveguide connection to the HPW-feed line. (c) Coupling efficiency spectrum and (d) 3D radiation pattern of HPRNA connected to the plasmonic waveguide. | | |

**S2.2. Comparison of the proposed HPRNA performance with previous works**

Here, the advantages of the proposed HPRNA with Si director by investigation of radiation characteristics such as efficiency (e), gain (G), type of radiation pattern and fabrication possibility have been explained. For proposing on-chip nano-antenna for wireless transmission link, the horizontal radiation pattern is necessary. In comparison to the patch nano-antenna with bidirectional vertical radiation pattern and 15 THz bandwidthS3, by utilizing dielectric director the problem of the tilted angle beam is solved and the bandwidth is enhanced to 35 THz. Moreover, the problem of multi‑bandwidth horn nano-antennaS4, which could not be used in multi-channel structures is solved. Compared to the super-mode nano-antennaS5 with the efficiency of close to 9%, the proposed nano‑antenna has an efficiency of more than 95%. Although the gain enhancement of HPRNA compared to V‑shaped antennaS6 is about 1 dB at 193.5 THz, the issue of necessity of having horizontal pattern is addressed. On the other hand, the gain of proposed HPRNA compared to the patchS3, hornS4, VivaldiS7, and bow-tieS8 antennas is improved 4.4, 5.3, 5, and 1.03 dB at 193.5 THz, respectively. Also, in comparison to the bow‑tie HPW-based nano-antennaS8 and W‑shaped nano-antennaS2, the obtained propagation length increases almost 10 times. Choosing the rectangular cross-section to design HPRNA is one of the most important superiorities compared to the circular oneS9 in order to reduce the fabrication process complexity and improve the propagation length more than 12 times. Another advantage of the proposed HPRNA is related to its aspect ratio in comparison to the previous proposed nano-antennasS2,S6 that leads to utilizing typical EBL technique to fabricate it instead of complicated X-ray method. All these points reviewed are summarized in Table S1.

Table S1. Comparison of the results of our work with previous works at 193.5 THz.

| **Lp (µm)** | **(dB)G** | **e (%)** | **Horizontal main lobe** | **Fabrication Possibility** | **Ref.** |
| --- | --- | --- | --- | --- | --- |
| 9 | 10 | 94.57 | Yes | Hard | [S2] |
| 70> | 5.6 | 83 | No | Medium | [S3] |
| 70> | 4.67 | - | No | Medium | [S4] |
| 4 | 9.38 | 9 | No | Medium | [S5] |
| 8 | 10 | 92.25 | Yes | Very hard | [S6] |
| 70> | 5 | - | No | Medium | [S7] |
| 9 | 8.97 | 86 | Yes | Medium | [S8] |
| 217 | 9.39 | 94 | No | Hard | [S9] |
| 9 | 10.6 | 74.81 | Yes | Complicated | [S10] |
| 94.4 | 10 | 95.5 | Yes | Simple | Our work |

**S3. HPW-based polarization beam splitter**

The transmission spectra of PBS with HPW input section excited by TM and TE modes are depicted in Fig. S6. The obtained results reveal that by stimulating different plasmonic modes through the HPW port, most power transmits by HPW and dielectric waveguide for TM and TE modes, respectively. In contrast to Fig S6, if dielectric waveguide is considered as an input port, most power will transfer by HPW and dielectric waveguide for TE and TM excited modes, respectively, which these results are demonstrated in Fig. S7.

| **(a)** | **(b)** |
| --- | --- |
|  |  |
| **Figure S6.** Transmission spectra of the proposed PBS with HPW input section excited by (a) TM and (b) TE modes. | |
| **(a)** | **(b)** |
|  |  |
| **Figure S7.** Transmission spectra of the proposed PBS with dielectric waveguide input section excited by (a) TM and (b) TE modes. | |

To depict the performance of the PBS, the critical parameters of insertion loss (IL) and crosstalk (CT) for different excited modes at the frequency of 193.5 THz for both proposed HPW‑based PBSs are listed in Table S2. Moreover, in order to show the benefits and drawbacks of the proposed HPW-based PBS, it is compared with previous published works by considering essential factors including foot-print, waveguide type, coupling length, output power, polarization performance and bandwidth, which are listed in Table S3. It is clear that the suggested HPW-based PBS not only has higher working bandwidth compared to other structures based on dielectric, plasmonic and grating waveguidesS11‑S22, but also it has the simplest and most flexible design among all grating‑based structures.

Table S2. Insertion loss and crosstalk of different modes at 193.5 THz.

| **Parameter** | **PBS with the HPW input section** | | **PBS with the DW input section** | |
| --- | --- | --- | --- | --- |
|  | **Port 2 (dB)** | **Port 3 (dB)** | **Port 2 (dB)** | **Port 3 (dB)** |
| $\text{IL}_{TE}^{TE}$ | -20.31 | -21.61 | -10.22 | -30.54 |
| $\text{IL}_{TE}^{TM}$ | -4.42 | -31.63 | -33.85 | -1.14 |
| $\text{IL}_{TM}^{TM}$ | -30.15 | -1.25 | -0.24 | -30.88 |
| $\text{IL}_{TM}^{TE}$ | -18.27 | -33.45 | -29.32 | -31.72 |
| $\text{CT}_{TE}^{HPW,DW}$ | - | -10.61 | -23.63 | - |
| $\text{CT}_{TM}^{HPW,DW}$ | -11.88 | - | - | -0.84 |

On the other hand, using HPW idea to design the PBS leads to the reduction of coupling length significantly, which results in providing a very compact PBS compared to plasmonic and dielectric waveguides-based structures. Also, the proposed PBSs are able to convert both TE and TM modes, while the previous PSBsS11-S15 are only able to convert one mode. In addition, significant transmission power is achieved at the output port compared to other PBSsS11, S15-S18. Therefore, the advantages of the proposed PBSs are high output power, short coupling length, compact size, and high bandwidth.

Table S3. Comparison of the proposed HPW-based PBS with previous published works.

| **Polarization performance** | **Output power (%)** | **Coupling length (µm)** | **Bandwidth**  **(nm)** | **Waveguide type** | **Foot-print**  **(m2µ)** | **Ref.** |
| --- | --- | --- | --- | --- | --- | --- |
| TE0-to-TM1 | 39.81 | 11 | 200 | PW | 11×0.8 | [S11] |
| TM0-to-TE­1 | 98.2 | 200 | - | DW | - | [S12] |
| TM0-to-TE­1 | 95.49 | 9 | 100 | DW | - | [S13] |
| TE0-to-TE­1 | 96.01 | 5.75 | 21 | GDW | 29×2 | [S14] |
| TE0-to-TE­1 | 89.12 | 12 | 23 | DW | - | [S15] |
| TM0/TE1-to-TE­1­/TM0 | 70.79 | 21 | 115 | GDW | - | [S16] |
| TM0/TE1-to-TE­1­/TM0 | 84.91 | 6.8 | 115 | GDW | - | [S17] |
| TM0/TE1-to-TE­1­/TM0 | 79.43 | 23 | 60 | GDW | - | [S18] |
| TM0/TE1-to-TE­1­/TM0 | 95 | 7.7 | 70 | HPW | - | [S19] |
| TM0/TE1-to-TE­1­/TM0 | 95.49 | 2.42 | 100 | HPW | 2.6×8.1 | [S20] |
| TM0/TE1-to-TE­1­/TM0 | 95 | 2.2 | 120 | HPW | 3.7×1.9 | [S21] |
| TM0/TE1-to-TE­1­/TM0 | 86.89 | 8.13 | 100 | DW | - | [S22] |
| TM0/TE1-to-TE­1­/TM0 | 95 | 2.25 | 303 | HPW | 2.9×4.45 | Our work |
| DW: Dielectric Waveguide, GDW: Grating Dielectric Waveguide, PW: Plasmonic Waveguide | | | | | | |

**S3.1. Controllable HPW-based PBS**

To implement a controllable HPW-based PBS, the idea of using multilayer graphene has been consideredto haveswitchable output ports by controlling graphene chemical potential. As demonstrated in Fig. S8a, eight graphene sheets are set inside the HSQ layer. According to Fig. S8b,c, the electric field distributions of the switchable HPW-based PBS at 193.5 THz disclose that the transmission power can be controlled by setting a suitable graphene chemical potential.

| **(a)** | | | |
| --- | --- | --- | --- |
| 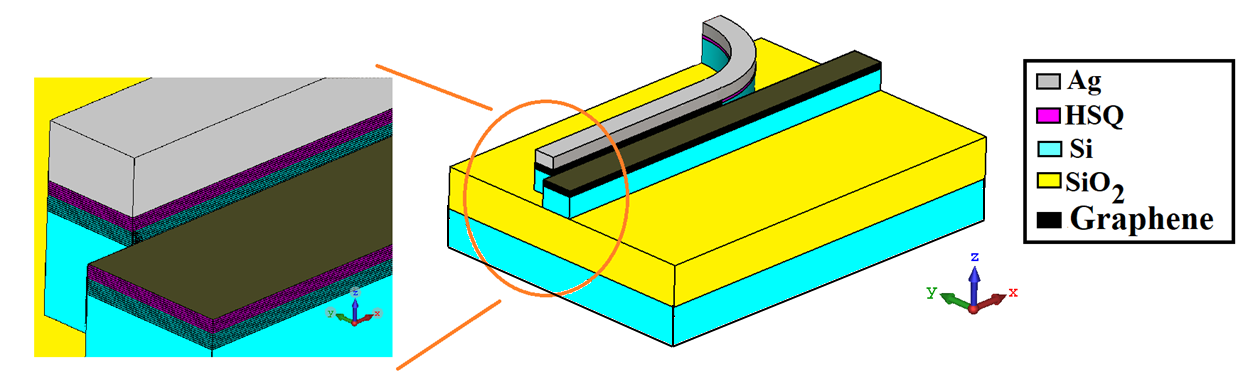 | | | |
| **(b)** | | **(c)** | |
| 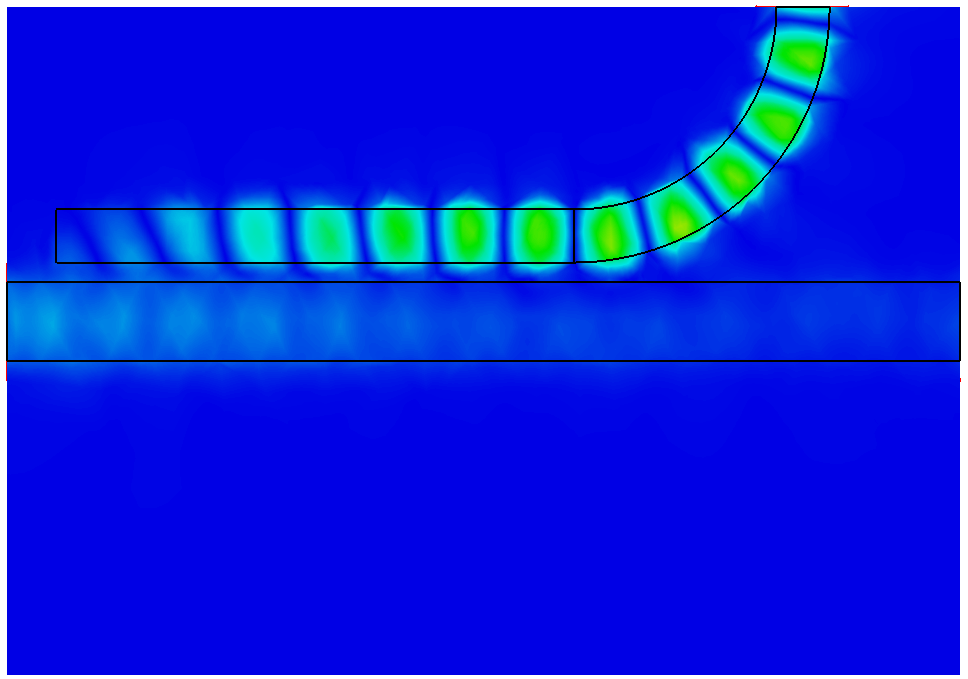 | 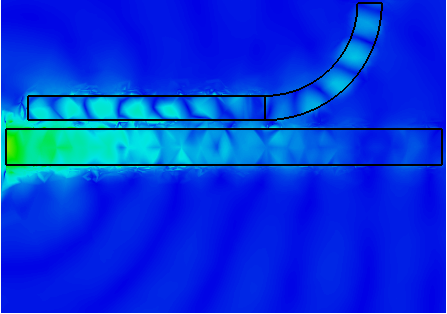 | | 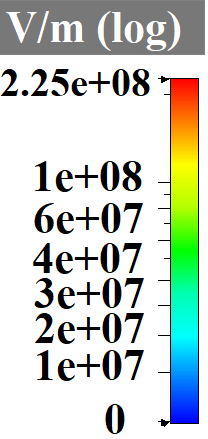 |
| **Figure S8.** (a) 3D schematic view of controllable HPW-based PBS. Electric field destitutions of controllable HPW-based PBS with dielectric waveguide input port for (b) $\mu_{c}=0 \text{eV}$ and (c) $\mu_{c}=0.51 \text{eV}$ at 193.5 THz. | | | |

Also, the transmission spectra of HPW output power for two values of $\mu_{c}=0 \text{eV}$ and $\mu_{c}=0.51 \text{eV}$ are plotted in Fig. S9. It can be seen that the transmission power is decreased drastically to less than 0.35% when $\mu_{c}=0.51 \text{eV}$. Also, by selecting $\mu_{c}=0 \text{eV}$, the transmission power is reduced from 76.85% to 70% in comparison to the obtained results of Fig. S7b due to the graphene intrinsic loss. Finally, the characteristics of our controllable HPW-based PBS are compared with previous published works in Table S4. It shows that when $\mu_{c}=0.51 \text{eV}$, the proposed structure is able to significantly reduce the output power close to zero value at 193.5 THz. Also, utilizing the idea of HPW for designing the PBS reduces the coupling length leading to a much more compact structural design than plasmonic and dielectric PBSs. Moreover, the bandwidth of the suggested PBS is increased at least more than 10 times compared to other controllable PBSs.

| **(a)** | **(b)** |
| --- | --- |
|  |  |
| **Figure S9.** Transmission spectra of HPW output power for the proposed controllable HPW-based PBS when $\mu_{c}=0 \text{eV}$ and (b) $\mu_{c}=0.51 \text{eV}$. | |

Table S4. Comparison of the proposed controllable HPW-based PBS with previous published works.

| **Ref.** | **Bandwidth (nm)** | **Coupling length (µm)** | **Output power at OFF state** | **Performance** | **Waveguide type** | |
| --- | --- | --- | --- | --- | --- | --- |
| [S23] | 86 | 11.1 | 10.88 | TM0/TE1-to-TE­1­/TM0 | DW-graphene |
| [S24] | 74 | 8.3 | 27.55 | TM0/TE1-to-TE­1­/TM0 | DW-graphene |
| [S25] | 35 | 100 | 37.15 | TM0/TE1-to-TE­1­/TM0 | PW-graphene |
| Our work | 303 | 2.25 | 0.25 | TM0/TE1-to-TE­1­/TM0 | HPW-graphene |

**S4. Controllable HPW-based 1×3 power splitter**

Here, the effects of structural parameters on the performance of HPW-based power splitter are studied. Since the losses of lateral branches are greater than the straight one, the width of lateral branches should be greater than the middle one. If the width of all three branches is considered the same, the output power of straight and lateral branches are 43.91%, 17.41%, and 17.41%, respectively. Table S5 shows the effect of changing the width of lateral branches $\left(W_{1}, W_{3}\right)$ on the power of output ports at 193.5 THz. By increasing the width of lateral branches, their transmission power gradually increases and then decreases. As a result, the best output power is obtained when $W_{1}=W_{3}=370 \text{nm}$. Also, by selecting $W_{1}=W_{3}=170 \text{nm}$, the output power of the straight branch is 43.91%, but the power of the other two branches is significantly reduced.

The transmission spectra of the structure for different values of *W*2 are plotted in Fig. S10. By increasing *W*2 from 166 to 206 nm, the output power of the straight branch is increased from 28.64% to 35.22% at 193.5 THz. However, the output power of lateral branches is decreased from 30.83% to 29.04%, hence the optimal response is obtained for *W*2 = 186 nm.

Another effective parameter for controlling the power of output ports is the distance between branches (*D*). As illustrated in Fig. S11, by increasing *D* from 245 to 255 nm, the output power is increased for all branches, then by changing *D* from 255 to 265 nm, the output power of the straight branch is enhanced. In return, the transmission power of lateral branches experiences a slightly decrease. For 265 nm<*D*<315 nm, the output power of three branches will not change significantly. However, by increasing *D* from 315 to 365 nm, the power of lateral branches is decreased significantly. Therefore, the optimal distance is considered to be *D* = 265 nm.

Table S5. Study of changing the width of lateral and straight branches in the power splitter at 193.5 THz.

| **Output3 (%)** | **Output2 (%)** | **Output1 (%)** | **Parameters** |
| --- | --- | --- | --- |
| 17.41 | 43.91 | 17.41 | $W_{1}=W_{3}=170\text{ nm}$ |
| 25.43 | 34.89 | 25.43 | $W_{1}=W_{3}=210\text{ nm}$ |
| 28.35 | 31.88 | 28.35 | $W_{1}=W_{3}=250\text{ nm}$ |
| 29.96 | 30.64 | 29.96 | $W_{1}=W_{3}=290\text{ nm}$ |
| 29.70 | 33.03 | 29.70 | $W_{1}=W_{3}=330\text{ nm}$ |
| 30.17 | 31.91 | 30.17 | $W_{1}=W_{3}=370\text{ nm}$ |
| 26.15 | 29.17 | 26.15 | $W_{1}=W_{3}=410\text{ nm}$ |

| **(a)** | **(b)** |
| --- | --- |
|  |  |
| 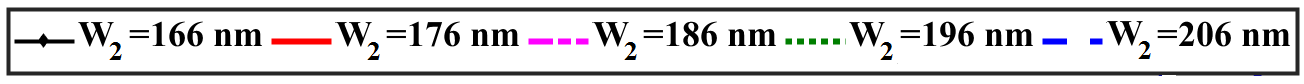 | |
| **Figure S10:** Transmission spectra of the (a) straight and (b) lateral branches versus different widths of the middle branch. | |


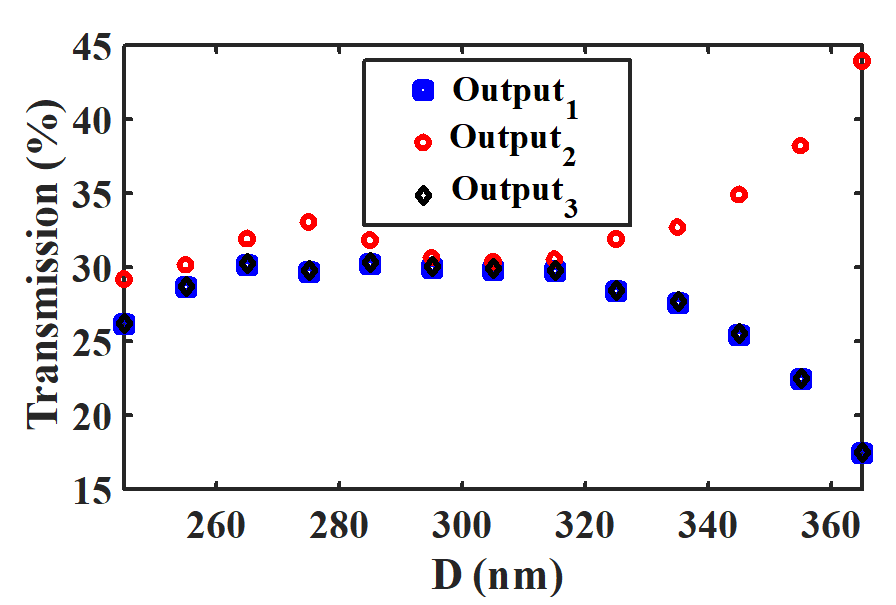


**Figure S11.** Transmission power of each branch of HPW-based power splitter versus different values of *D* at 193.5 THz.

The effect of graphene layers on the transmitted power to the output ports at 193.5 THz is depicted in Table S6. By increasing the graphene layers from 1 to 6, the output power of lateral branches is decreased. Also, the output power of middle branch is reduced by increasing the graphene layers from 1 to 9. Consequently, increasing the number of layers beyond eight layers has little effect on the amount of received power at each branch and only increases the fabrication complexity.

Table S6. Effect of number of graphene layers on the output powers at 193.5 THz, when $\mu_{c1}=\mu_{c3}=0 \text{eV}$ and $\mu_{c2}=0.51 \text{eV}$.

| **9** | **8** | **7** | **6** | **5** | **4** | **3** | **2** | **1** | **No. Graphene layers** |
| --- | --- | --- | --- | --- | --- | --- | --- | --- | --- |
| 29.85 | 29.87 | 27.35 | 25.08 | 26.1 | 27.12 | 28.02 | 28.88 | 29.35 | **Output1 (%)** |
| 0.135 | 0.14 | 2.62 | 4.84 | 7.18 | 9.88 | 14.56 | 19.24 | 25.66 | **Output2 (%)** |
| 29.85 | 29.87 | 27.35 | 25.08 | 26.1 | 27.12 | 28.02 | 28.88 | 29.35 | **Output3 (%)** |

**S5. HPW-based coupler**

The most important structural parameters for analysis of HPW-based coupler are *L*1 and *L*2, which their effects on the output powers at 193.5 THz are demonstrated in Table S7.

Table S7. The effect of coupling length on the output power at 193.5 THz.

| 3350=*L*­1  3200=*L*2 | 3320=*L*­1  3170=*L*2 | 3290=*L*­1  3140=*L*2 | 3260=*L*­1  3110=*L*2 | 3230=*L*­1  3080=*L*2 | 3200=*L*­1  3050=*L*2 | 3170=*L*­1  3020=*L*2 | 3140=*L*­1  2990=*L*2 | **Coupling length (nm)** |
| --- | --- | --- | --- | --- | --- | --- | --- | --- |
| 0.36 | 0.37 | 0.24 | 0.44 | 0.031 | 0.17 | 0.30 | 0.43 | **P­2 (%)** |
| 75.77 | 76.96 | 77.15 | 77.62 | 88.13 | 77.59 | 77.18 | 77.60 | **P3 (%)** |
| 0.009 | 0.009 | 0.008 | 0.008 | 0.005 | 0.009 | 0.0098 | 0.013 | **P4 (%)** |

**S6. Circular HPW-based filter and refractive index sensor**

Figure S12 shows the schematic view of HPW-based ring resonator with rectangular waveguide. In the following, the effect of some parameters on the performance of circular HPW-based filter will be investigated. Figure S13a depicts the effect of changing the inner radius of ring resonator (*R*1) on the transmission spectrum. By increasing *R*1 from 240 to 290 nm, the resonance frequency is shifted to lower frequencies. It shows that to design a narrow‑band filter with the desired resonance frequency, only the resonator radius should be modified. In addition, as the radius increases, the second mode appears for some radii. By increasing *R*1 from 240 to 270 nm, the ER is improved from 15 to 40.5 dB and then it is decreased for more increase of *R*1.

Studying the effect of *R*1 on the resonance frequency, FWHM, and ER of circular HPW-based filter is illustrated in Table S8. As it is mentioned earlier, one of the reasons for using circular HPW is to increase *g*, which significantly reduces the fabrication complexity. Also, as demonstrated in Fig. S13b, controlling *g* plays an important role in the value of ER due to its effect on the coupling efficiency between the ring and waveguide.

| **(a)** | **(b)** | |
| --- | --- | --- |
| 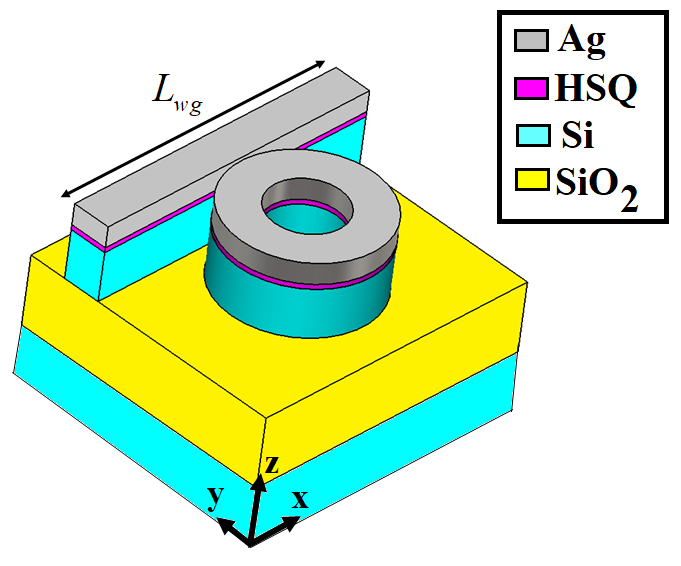 | 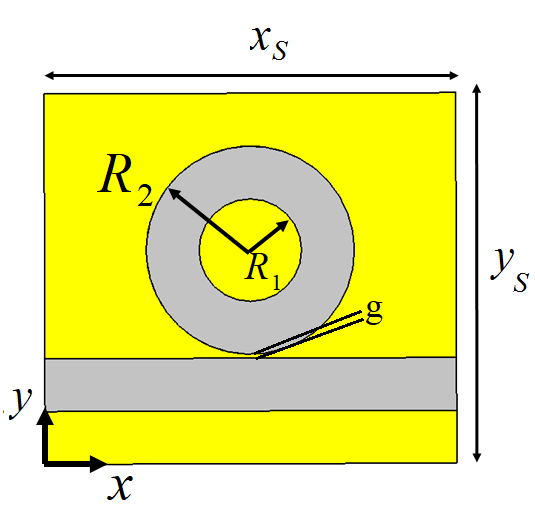 | |
| **Figure S12.** (a) 3D schematic and (b) top views of HPW-based filter with rectangular HPW. The geometrical parameters are *g*=10 nm, *Lwg*=2100 nm, *R*1=270nm, *R*2=520 nm, and $x_{s}\times y_{s}=2100\times1890 \text{nm}^{2}$ | | |
| **(a)** | | **(b)** | |
|  | |  | |
| **Figure S13.** Transmission spectra of circular HPW-based filter for different values of (a) *R*1 and (b) *g*. | | | |

Table S8. Effect of *R*1 on the resonance frequency, FWHM, and ER of circular HPW-based filter.

| ***R*­1 (nm)** | ***f* (THz)** | **FWHM (nm)** | **ER (dB)** |
| --- | --- | --- | --- |
| 240 | 202.74 | 13 | 15 |
| 250 | 199.62 | 12 | 19.6 |
| 260 | 196.54 | 6 | 30 |
| 270 | 193.5 | 5 | 40.5 |
| 280 | 190.56 | 9 | 29 |
| 290 | 187.72 | 12 | 26.5 |

**References**

S1. Krasavin, A. V. & A.V. Zayats. Active nanophotonic circuitry based on dielectric-loaded plasmonic Waveguides. *Adv. Opt. Mat*. **3**,1662-1690 (2015).

S2. Khodadadi, M., Nozhat, N. & Moshiri, S. M. M. Analytic approach to study a hybrid plasmonic waveguide-fed and numerically design a nano-antenna based on the new director. *Opt. Express* **28**, 3305-3330 (2020).

S3. Youseﬁ, L. & Foster, A. C. Waveguide-fed optical hybrid plasmonic patch nano-antenna. *Opt. Express* **20**, 18326–18335 (2012).

S4. Nourmohammadi, A. & Nikoufard, M. Ultra-wideband photonic hybrid plasmonic horn nanoantenna with SOI configuration. *Silicon* **12**, 193-198 (2019).

S5. Khodadadi, M. & Nozhat, N. Theoretical analysis of a super-mode waveguide and design of a complementary triangular hybrid plasmonic nano-antenna. *IEEE J. Sel. Top. Quantum Electron*. **27**, 4600210 (2021).

S6. Khodadadi, M., Nozhat, N. & Moshiri, S. M. M. A High gain and wideband on-chip hybrid plasmonic V‑shaped nano-antenna. *J. Opt.* **22**, 035005 (2020).

S7. Saad-Bin-Alam, M., Khalil, M. I., Rahman, A. & Chowdhury, A. M. Hybrid plasmonic waveguide fed broadband nanoantenna for nanophotonic applications. *IEEE Photon. Technol. Lett*. **27**, 1092-1095 (2015).

S8. Nikoufard, M., Nourmohammadi, A. & Esmaeili, S.; Hybrid plasmonic nanoantenna with the capability of monolithic integration with laser and photodetector on InP substrate. *IEEE Trans. Antennas Propag*. **66**, 3-8 (2018).

S9. Khodadadi, M., Nozhat, N. & Moshiri, S. M. M. Theoretical analysis of a circular hybrid plasmonic waveguide to design a hybrid plasmonic nano-antenna. *Sci.‎ Rep*, **10**, 15122 (2020).

S10. Khodadadi, M., Nozhat, N. & Moshiri, S. M. M. [Theoretical analysis of a graphene quantum well hybrid plasmonic waveguide to design an inter/intra-chip nano-antenna](https://scholar.google.com/citations?view_op=view_citation&hl=en&user=U7FRi-QAAAAJ&sortby=pubdate&citation_for_view=U7FRi-QAAAAJ:Tyk-4Ss8FVUC). *Carbon* **189**, 443-458 (2022).

S11. Qi, Z. *et al*. Fano resonances in ultracompact silicon-on-insulator compatible integrated photonic-plasmonic hybrid Circuits. *Adv. Optic. Mat.* **5**, 1700304 (2017).

S12. Dai, D. & Zhang, M. Mode hybridization and conversion in silicon-on-insulator nanowires with angled sidewalls. *Opt. Express* **23**, 32452–32464 (2015).

S13. Guan, H. *et al.* Ultracompact silicon-on-insulator polarization rotator for polarization-diversiﬁed circuits. *Opt. Lett.* **39**, 4703-4706 (2014).

S14. Wang, H. *et al*. Compact silicon waveguide mode converter employing dielectric metasurface structure. *Adv. Opt. Mater*. **7**, 1801191 (2019).

S15. Perez-Galacho, D. *et al*. Mode converters based on periodically perturbed waveguides for mode division multiplexing. *Proc. SPIE* **10686**, (2018).

S16. Liu, L., Deng, Q. & Zhou, Z. Manipulation of beat length and wave length dependence of a polarization beam splitter using a subwavelength grating. *Opt. Lett*. **41**, 5126–5129 (2016).

S17. Xu, Y. & Xiao, J. Compact and high extinction ratio polarization beam splitter using subwavelength grating couplers. *Opt. Lett*. **41**, 773‑776 (2016).

S18. Chen, S., Wu, H. & Dai, D. High extinction-ratio compact polarization beam splitter on silicon. *Electron. Lett.* **52**, 1043–1045 (2016).

S19. Bai, B., Liu, L. & Zhou, Z. Ultracompact, high extinction ratio polarization beam splitter-rotator based on hybrid plasmonic-dielectric directional coupling. *Opt. Lett.* **42**, 4752-4755 (2017).

S20. Bai, B., Deng, Q. & Zhou, Z. Plasmonic-assisted polarization beam splitter based on bent directional coupling. *IEEE Photo. Tech. Lett.* **29**, 599-602 (2017).

S21. Guan, X., Wu, H., Shi, Y., Wosinski, L. & Dai, D. Ultracompact and broadband polarization beam splitter utilizing the evanescent coupling between a hybrid plasmonic waveguide and a silicon nanowire. *Opt. Lett*. **38**, 3005-3008 (2013).

S22. Hsu, C. W., Chang, T. K., Chen, J. Y. & Cheng, Y. C. 8.13 μm in length and CMOS compatible polarization beam splitter based on an asymmetrical directional coupler. *Appl. Opt*. **55**, 3313-3318 (2016).

S23. Zhang, T., Ke, X., Yin, X., Chen, L. & Li, X. Graphene-assisted ultra-compact polarization splitter and rotator with an extended bandwidth. *Sci. Rep*. **7**, 12169 (2017).

S24. Zhang, T., Yin, X., Chen, L. & Li, X. Ultra-compact polarization beam splitter utilizing a graphene-based asymmetrical directional coupler. *Opt. Lett*. **41**, 356-359 (2016).

S25. Shin, J. S., Kim, J. S. & Kim, J. T. Graphene-based hybrid plasmonic modulator. *J. Opt.* **17**, 125801 (2015).
